# Nonequilibrium Electrical Double Layer Effects on Unilateral Peak Suppression in Cyclic Voltammetry

**DOI:** 10.1021/acselectrochem.5c00491

**Published:** 2026-04-10

**Authors:** Yupeng Qin, Zhangquan Peng, Jun Huang

**Affiliations:** 1 Laboratory of Advanced Spectro-electrochemistry and Li-Ion Batteries, Dalian Institute of Chemical Physics, Chinese Academy of Sciences, Dalian 116023, China; 2 University of the Chinese Academy of Sciences, Beijing 100049, China; 3 Institute of Energy Technologies, IET-3: Theory and Computation of Energy Materials, Forschungszentrum Jülich GmbH, Jülich 52425, Germany; 4 Faculty of Georesources and Materials Engineering, RWTH Aachen University, Aachen 52062, Germany

**Keywords:** cyclic voltammetry, nonequilibrium electrical double layer effects, Poisson−Nernst−Planck equation, Frumkin correction, electrode-reactant repelling

## Abstract

While equilibrium electrical double layer (EDL) effects on steady-state voltammetry have been studied, nonequilibrium transient EDL effects on cyclic voltammetry (CV) remain less understood. Herein, we report a combined theoretical and experimental study of nonequilibrium EDL effects on CV, focusing on their unequal influence over the cathodic and anodic peaks. The experiments are analyzed with the aid of a theoretical model coupling the Poisson–Nernst–Planck (PNP) theory and various electrode kinetic theories that can describe interfacial electron transfer reaction, EDL formation, and macroscopic mass transport in a unified framework. Consistent with experiments, the model predicts suppressed cathodic peaks for the CV of the [Fe­(CN)_6_]^3–/4–^ redox reaction and correlates them with the spatiotemporal distributions of electrical potential and reactant concentration in the EDL. The model is then utilized as an efficient computational tool for exploration in the vast parametric space, identifying parametric regions where pronounced nonequilibrium EDL effects could be observed in CV. A regime diagram of nonequilibrium EDL effects on CV is calculated and validated partly with experiments. In general, the unilateral suppression of CV peaks is more pronounced when the reactive ion is repelled from the like-charged electrode surface at a higher scanning rate.

## Graphical abstract

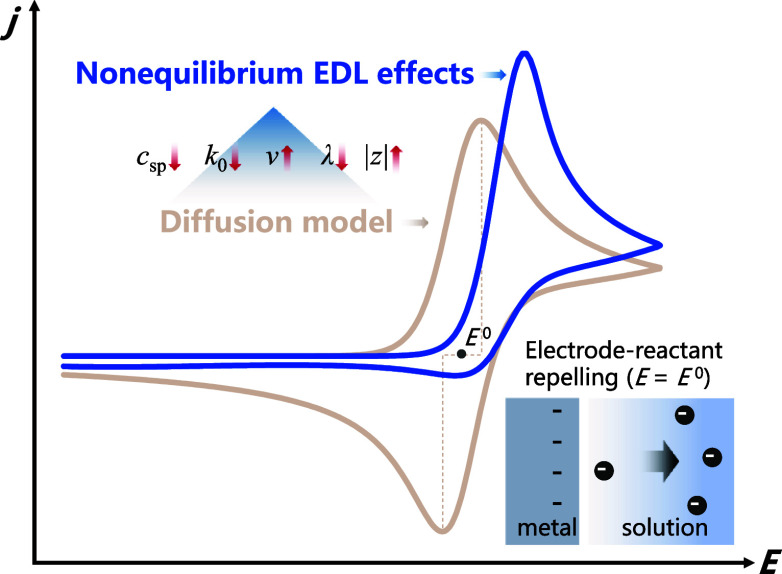

## Introduction

A classical problem in electrode kinetics concerns how multiscale mass transport influences interfacial charge transfer. In general, multiscale mass transport determines the local reactant concentration and overpotential, which together influence the interfacial charge transfer rate. The interfacial charge transfer rate, in return, shapes mass transport in both the nanoscopic EDL and the macroscopic solution phase. While macroscopic mass transport in the electroneutral solution is well understood, nanoscopic mass transport in the EDL at the electrode–electrolyte interface remains a topic of active research.^1–4^

In 1933, Frumkin first recognized the roles of EDL in electrode kinetics by influencing the concentrations and electric potential at the electrode interface. He corrected the Erdey-Grúz–Volmer theory,^5^ also known as the Butler–Volmer theory, by incorporating the equilibrium EDL theory, successfully explaining the cation effects on the hydrogen evolution reaction on mercury electrodes.^6^ In 1949, Kryukova observed a dramatic decrease of reduction current of peroxydisulfate anions on mercury when the electrode potential is shifted negative of the potential of zero charge (PZC, *E*_pzc_).^7^ Two years later, Frumkin and Florianovich interpreted this phenomenon utilizing the Erdey-Grúz–Volmer theory with Frumkin corrections.^8^ Subsequently, Gierst et al., Fawcett and Markušová, and Weaver *et al*. applied, with necessary modifications, the Frumkin correction to the oxidation of europium­(II) cations,^9–11^ reduction of chromium­(III) complexes,^12,13^ and metallocene couples.^14^ Tsirlina and coworkers made notable extensions to the Frumkin corrections. Nazmutdinov and Tsirlina released the restriction that electron transfer reaction occurs on a reaction plane in the EDL; instead, they considered that electron transfer reactions occur in a spatially extended volume, and the reaction volume is (potential) charge-dependent.^15^ More recently, Tsirlina *et al*. recognized the limitations of Erdey-Grúz–Volmer theory in describing electron transfer reactions in a wide potential range and incorporated the Frumkin corrections into the Marcus theory.^16,17^ Out of this improvement, corrected Marcus plots were developed to describe EDL effects on electrode reactions in an extended potential range.

While most studies on EDL effects have assumed an equilibrium structure of the EDL, Levich was the first to discuss nonequilibrium EDL effects in 1949,^18^ *cf.*, a recent commentary article.^19^ He recognized that the EDL deviates from equilibrium in the presence of faradaic reactions. His theoretical analysis reveals that the EDL deviates more significantly from equilibrium when the reactant is repelled by a like-charged electrode surface. On the contrary, nonequilibrium EDL effects are minimal in the case where the reactant is attracted to an oppositely charged electrode surface. He *et al.*,^20^ Norten *et al.*,^21^ and Dickinson and Compton^22^ adopted Levich’s idea to understand nonequilibrium EDL effects on the steady-state voltammetry of nanoelectrodes. At nanoelectrodes, the characteristic length of the diffusion layer is comparable to the thickness of the EDL, resulting in EDL effects on the diffusion limiting current.^20–24^ Specifically, at nanoelectrodes with a radius smaller than 20 nm, the diffusion limiting current deviates markedly from the value predicted by classical transport models even for fast electron transfer systems such as hexaammineruthenium-III ([Ru­(NH_3_)_6_]^3+^) and hexachloroiridate-IV ([IrCl_6_]^2–^).^25–27^ As for more sluggish kinetics systems like [Fe­(CN)_6_]^3–/4–^, the EDL effects on the diffusion limiting current can be observed for much larger electrodes up to a radius of 1000 nm.^26^

In steady-state voltammetry, the local reaction conditions in the EDL are state functions of the electrode potential while being independent of the path of reaching the electrode potential. In contrast, in transient voltammetry, the EDL at a given electrode potential depends on the whole path leading to this electrode potential. Nonequilibrium EDL effects on transient voltammetry have been investigated in the recent theoretical study by Levey *et al.* ^28^ It was found that the repelling force of the charged surface on reactive ions diminishes the peak currents and increases peak separation, whereas the opposite occurs when ions are attracted by the charged surface. Additionally, the intensity of nonequilibrium EDL effects depends on both the kinetic constant and the scanning rate.

The implications of the model of Levey *et al.* regarding EDL effects on transient CV remain incompletely explored. An important observation in CV measurements is that the magnitudes of cathodic and anodic peaks are markedly unequal even in the absence of subsequent irreversible chemical reactions—a usual cause of suppressed peaks in CV.^29^ For instance, CV profiles measured for [Fe­(CN)_6_]^3–/4–^ redox reaction without any supporting electrolyte show a severely suppressed cathodic peak.^30^ In accordance with experimental observations, the simulations by Levey *et al.* also showed significantly broader cathodic peaks than anodic peaks when the reactive ions are repelled by the charged surface.^28^ While the influence of the EDL on the steady-state polarographic behavior is well documented, its manifestation in transient voltammetry is not well understood. Specifically, the origin of unilateral peak suppression remains elusive despite the established role of the EDL.

In this study, we aim to understand nonequilibrium EDL effects on the transient CV of simple electrochemical reactions, specifically the origin of suppressed CV peaks, by combining theoretical modeling and experiments. As an improvement over the model used in the work of Levey *et al*., the Marcus–Hush–Chidsey theory is used here to describe the electron transfer, allowing us to explore the influence of the solvent reorganization energy on nonequilibrium EDL effects on the one hand and test the robustness of the theoretical findings against the variety in descrbing electron transfer on the other. In addition, the model is then employed to analyze the nonequilibrium EDL effects across a range of parameters, including the supporting electrolyte, charge on reactive ions, potential of zero charge, kinetic constant of redox reactions, and scanning rate. We further tested the model-based results with experiments.

## Methods

### Experiments

#### Solution Preparation

A 1 mM potassium ferricyanide (K_4_Fe­(CN)_6_, ≥98%, Adamas-beta) aqueous solution was prepared by dissolving 7.3 mg K_4_Fe­(CN)_6_ in 20 mL deionized water. Then, 6.1 and 61.2 mg of sodium perchlorate (NaClO_4_ ≥99.99%, Aladdin) were dissolved in 5 mL of the above solutions to get solutions with 10 and 100 mM supporting electrolyte, respectively. NaClO_4_ was chosen as the supporting salt because perchlorate ions do not exhibit significant specific adsorption. One millimolar solutions of the other redox couples were prepared similarly using 6.6 mg potassium ferrocyanide (K_3_[Fe­(CN)_6_], ≥99.5%, Aladdin)) and 6.2 mg hexaammineruthenium-III chloride ([Ru­(NH_3_)_6_]­Cl_3_, Alfa Aesar), respectively. All solutions were prepared with deionized water (18.2 MΩ·cm at 25 °C) from the HHitech purification system. The solutions were deoxygenated using high-purity argon.

#### CV Measurement

The CV measurements were conducted on a three-electrode system by using an electrochemical workstation (SP-300, BioLogic). The gold working electrode with a diameter of 2 mm (CH Instruments, Inc.) was polished with 1 μm aluminum oxide and then ultrasonically cleaned with deionized water. During all measurements, the working electrode was positioned facing downward. A saturated calomel electrode (SCE, Tianjin Ada Hengsheng Technology Co., Ltd.) was used as the reference electrode. To reduce the interference of the ions leaking from the SCE on the dilute electrolyte, the SCE was placed in a fritted glass tube filled with the electrolyte. Platinum wires were used as the counter electrode. All experiments were performed in the cell at room temperature. The scanning rate was 10 mV/s unless otherwise noted. To prevent signal distortion resulting from excessive compensation, the experimental ohmic compensation ratio was set at 85%. According to the expression derived by Bond and Feldberg,^32^ the difference in peak potentials between 85% and 100% compensation ratios is less than 5 mV under the conditions examined here. Therefore, the difference in the compensation ratio between 85% and 100% shall have a minimal effect on CV profiles under the examined cases.

### Models

A one-dimensional (1D) model was employed for studying nonequilibrium EDL effects on transient CV profiles, considering the six orders of magnitude difference between the electrode size and the EDL thickness. It should be noted that the 1D treatment is not always safe for macroelectrodes. A recent study has shown that the radial diffusion of the macroelectrode edge causes the peak current response to deviate from the description of the Randles–Ševčík equation.^33^ While the edge effects interfere with peak current magnitudes, their influence on asymmetric peak suppression is assumed to be minor. Therefore, a 1D model is used to simplify the theoretical analysis. We considered an outersphere, one-electron-transfer reaction without specific ionic adsorption. The Nernst–Planck equation was used to describe mass transport:

$$\frac{\partial c_{i}}{\partial t}=-\frac{\partial J_{i}}{\partial x},J_{i}=-D_{i}\frac{\partial c_{i}}{\partial x}-\frac{z_{i}F}{RT}D_{i}c_{i}\frac{\partial \phi }{\partial x}$$ 1

where *J*_*i*_, *D*_*i*_, *z*_*i*_, and *c*_*i*_ are, respectively, the flux, diffusion coefficient, charge, and concentration of species *i*. *F* is the Faraday constant, *R* is the gas constant, *T* is the temperature, and ϕ is the electric potential.

The electric field and ion densities are coupled via Poisson equation:

$$\epsilon_{s}\frac{\partial^{2}\phi }{\partial x^{2}}=-F\sum z_{i}c_{i}$$ 2

where ϵ_s_ is the permittivity of the solvent, which is taken as 78.5 ϵ_0_ for water in our work. ϵ_0_ is the permittivity of a vacuum.

On the electrode surface, the total current is the sum of the charge transfer current (*j*_ct_) and electrical double layer current (*j*_dl_):

$$j=j_{\mathrm{ct}}+j_{\mathrm{dl}}$$ 3

*j*_ct_ is related to the interfacial flux of the oxidant (*J*_0_) and reductant (*J*_r_):

$$\frac{j_{\mathrm{ct}}}{F}=J_{o}=-J_{r}=k_{\mathrm{ox}}^{m}c_{r}-k_{\mathrm{red}}^{m}c_{o}$$ 4

where *k*_ox_^*m*^ and *k*_red_^*m*^ are the rate constants for oxidation and reduction, respectively. *c*_r_ and *c*_0_ are the local concentrations of the reductant and oxidant, respectively. The superscript *m* is used to distinguish the Erdey-Grúz–Volmer kinetics (*m* = EGV) and Marcus–Hush–Chidsey kinetics (*m* = MHC). The EGV kinetics are expressed as

$$k_{\mathrm{ox}}^{\mathrm{EGV}}=k_{0}\exp\left(\left(1-\alpha \right)\frac{F}{RT}\eta_{\mathrm{eff}}\right)$$ 5

$$k_{\mathrm{red}}^{\mathrm{EGV}}=k_{0}\exp\left(-\alpha \frac{F}{RT}\eta_{\mathrm{eff}}\right)$$ 6

$$\eta_{\mathrm{eff}}=E-E^{0}-\phi_{H}+\beta \Delta \phi_{\mathrm{ohm}}$$ 7

where *k*_0_ is the standard kinetic constant and α is the transfer coefficient, which is assumed to be 0.5 in this work. The effective overpotential (η_eff_) is *E* – *E* ^0^ – ϕ_H_ + βΔϕ_ohm_, with *E* being the applied electrode potential, *E* ^0^ the formal potential, and ϕ_H_ the potential at the Helmholtz plane (HP). An explicit consideration of ϕ_H_ in η_eff_ is the essence of the Frumkin corrections. βΔϕ_ohm_ is introduced here to compensate for ohmic polarization in the electrolyte solution. β is the compensation ratio. The ohmic potential drop Δϕ_ohm_ is given by

$$\Delta \phi_{\mathrm{ohm}}=-{\left(\frac{\partial \phi }{\partial x}\right)}_{x=L}L$$ 8

where *L* is the distance from the working electrode to the reference electrode.

An improved description of electrode kinetics is based on the MHC theory, which expresses the rate constants as

$$\kappa_{\mathrm{ox}}=\frac{k_{B}T}{h}\int_{-\infty }^{\infty }\rho (1-f(\varepsilon ))(4\pi k_{B}T\lambda {)}^{-1/2}\exp\left(-\frac{(\varepsilon -e_{0}\eta_{\mathrm{eff}}+\lambda {)}^{2}}{4k_{B}T\lambda }\right)d\varepsilon$$ 9

$$\kappa_{\mathrm{red}}=\frac{k_{B}T}{h}\int_{-\infty }^{\infty }\rho f(\varepsilon )(4\pi k_{B}T\lambda {)}^{-1/2}\exp\left(-\frac{(\varepsilon -e_{0}\eta_{\mathrm{eff}}-\lambda {)}^{2}}{4k_{B}T\lambda }\right)d\varepsilon$$ 10

$$f\left(\varepsilon \right)=\frac{1}{1+\exp\left(\frac{\varepsilon }{k_{B}T}\right)}$$ 11

where κ_ox_ and κ_red_ are the oxidation and reduction rate constants.^34,35^ ρ is the effective density of electronic states, which is assumed to be a constant, namely, the wide-band approximation is used here.^36^ *f*(ε) is the Fermi–Dirac function. *e*_0_ is the elementary charge, *k*_B_ is the Boltzmann constant, *h* is the Planck constant, and λ is the solvent reorganization energy. Referenced to *k*_0_ corresponding to κ_ox_ and κ_red_ at η_eff_ = 0, the rate constants are rephrased as^37,38^

$$k_{\mathrm{ox}}^{\mathrm{MHC}}=k_{0}\frac{\kappa_{\mathrm{ox}}}{\kappa_{\mathrm{ox}}(\eta =0)}=k_{0}\frac{\int_{-\infty }^{\infty }(1-f(\varepsilon ))\exp\left(-\frac{(\varepsilon -e_{0}\eta_{\mathrm{eff}}+\lambda {)}^{2}}{4k_{B}T\lambda }\right)d\varepsilon }{\int_{-\infty }^{\infty }(1-f(\varepsilon ))\exp\left(-\frac{(\varepsilon +\lambda {)}^{2}}{4k_{B}T\lambda }\right)d\varepsilon }$$ 12

$$k_{\mathrm{red}}^{\mathrm{MHC}}=k_{0}\frac{\kappa_{\mathrm{red}}}{\kappa_{\mathrm{red}}(\eta =0)}=k_{0}\frac{\int_{-\infty }^{\infty }f(\varepsilon )\exp\left(-\frac{(\varepsilon -e_{0}\eta_{\mathrm{eff}}-\lambda {)}^{2}}{4k_{B}T\lambda }\right)d\varepsilon }{\int_{-\infty }^{\infty }f(\varepsilon )\exp\left(-\frac{(\varepsilon -\lambda {)}^{2}}{4k_{B}T\lambda }\right)d\varepsilon }$$ 13

where the integration range of ε can be safely limited to a finite range from −1 to 1 eV.

The electric potential distribution is schematically shown in Figure 1. According to the Gauss’s law, metal surface charge density (σ_M_) is given by

$$\sigma_{M}=-\epsilon_{s}{\left(\frac{\partial \phi }{\partial x}\right)}_{x={\mathrm{HP}}^{+}}=-\epsilon_{H}{\left(\frac{\partial \phi }{\partial x}\right)}_{x={\mathrm{HP}}^{-}}$$ 14

where *x* = HP^+^ (*x* = HP^–^) means that *x* approaches HP from the right (left) side, respectively. ϵ_H_ is the permittivity of the region between the electrode surface and the HP (inner layer). As there is no charge in the inner layer, the potential distribution is linear therein:

$$-\epsilon_{s}{\left(\frac{\partial \phi }{\partial x}\right)}_{x={\mathrm{HP}}^{+}}=\epsilon_{H}\frac{\left(\phi_{M}-\chi_{M}\right)-\phi_{H}}{\delta_{H}}=\epsilon_{H}\frac{\left(E-E_{\mathrm{pzc}}\right)-\phi_{H}}{\delta_{H}}$$ 15

where ϕ_M_ and χ_M_ are the inner potential and surface potential of the metal, respectively. ϕ_M_ – χ_M_ is equivalent to *E* – *E*_pzc_ (see details in Section S1 of the Supporting Information). We use δ_H_ = 0.6 nm, the typical radius of hydrated complex ions.

**Figure 1:**
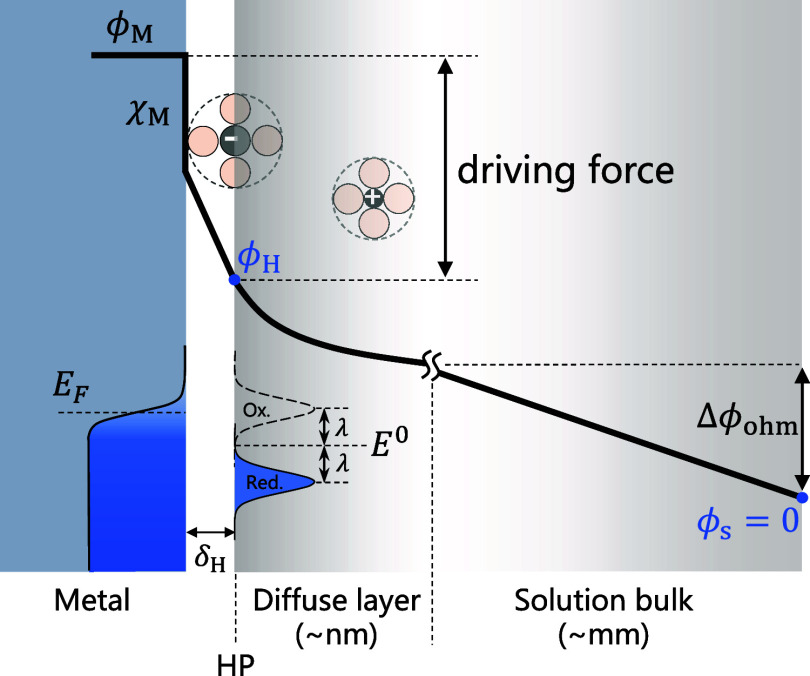
Schematic diagram of the electric potential distribution. The electric potential difference between the inner potential of the metal (ϕ_M_) and that of the bulk solution (ϕ_s_) is composed of the surface potential (χ_M_), a linear potential distribution in the inner layer, the potential drop in the diffuse layer, and the ohmic drop (Δϕ_ohm_). The difference between ϕ_M_ and the potential at the HP (ϕ_H_) is a key variable in electrode kinetics. δ_H_ is the thickness of the inner layer and is taken as the radius of the solvated anions. The bulk solution is chosen as the potential reference, ϕ_s_ = 0. The electron transfer is described by the MHC theory, with *E*_F_ being the Fermi level, *E* ^0^ the formal potential of the redox couple, and λ the solvent reorganization energy.

The electrical double layer current is obtained by

$$j_{\mathrm{dl}}=\frac{\partial \sigma_{M}}{\partial t}$$ 16

In addition to eq 14, another frequently used formula for *σ*_M_ is^39,40^

$$\sigma_{M}=-\int F\underset{i}{\sum }z_{i}c_{i}dx$$ 17

expressing the electrode surface charge as the negative of the total diffuse charge in the solution. Zhang *et al.* pointed out that eqs 14 and 17 are equivalent only if the electric field vanishes in the bulk solution.^27^ When there is a finite electric field in the solution, such as under nanoconfined conditions or under a high-frequency AC stimulus, σ_M_ is not equal to the total diffuse charge. Since a nonzero electric field exists in the bulk solution due to the ohmic resistance, we should use eq 14 to calculate σ_M_.

In the bulk solution, the concentration is equal to the bulk concentration, *c*_*i*_^b^. The electric potential is set as zero:

$$c_{i}=c_{i}^{b}$$ 18

$$\phi_{s}=0$$ 19

The supporting ions are monovalent. The parameters of the redox couple in the model correspond to [Fe­(CN)_6_]^3–/4–^ and are listed in Table 1. The CV simulation is implemented by imposing the *E* of triangular waves at the electrode boundary. The model is calculated using COMSOL Multiphysics 6.2. All governing equations and boundary conditions are expressed in dimensionless parameters, and the model is constructed using coefficient-form partial differential equations. A step-by-step tutorial of implementation of the model is provided in Section S6 of the Supporting Information.

**Table 1:** Values of the Model Parameters Used in the Base Case

symbol	meaning	value or range	note
*D*_0_	diffusion coefficient of the oxidant	7.0 × 10^–10^ m^2^/s	refs 30 and 41
*D*_r_	diffusion coefficient of the reductant	7.0 × 10^–^^10^ m^2^/s	refs 30 and 41
*D*_p_	diffusion coefficient of the positive supporting ions	2.0 × 10^–9^ m^2^/s	refs 23 and 30
*D*_n_	diffusion coefficient of the negative supporting ions	2.0 × 10^–9^ m^2^/s	refs 23 and 30
*k*_0_	kinetic constant	0.001–0.1 cm/s	refs 42–46
ϵ_H_	permittivity of the inner layer	4.0ϵ_0_	estimated
ϵ_s_	permittivity of the solution	78.5ϵ_0_	based on water
δ_H_	thickness of the inner layer	0.6 nm	estimated
α	transfer coefficient	0.5	estimated
λ	solvent reorganization energy	1.2 eV	ref 47
*T*	temperature	298.15 K	

## Results and Discussion

### Experimental Observation of Suppressed Cathodic Peaks and Four Possible Causes

In line with the literature,^30^ we observe a severely suppressed cathodic peak of the [Fe­(CN)_6_]^3–/4–^ redox reaction on gold electrode at low ionic strength. As illustrated in Figure 2, the CV curve measured on gold with 1 mM K_4_Fe­(CN)_6_ in a 100 mM NaClO_4_ aqueous solution exhibits well-defined reversibility. As the concentration of NaClO_4_ decreases, however, the anodic peak shifts to a higher potential, and the intensity of the cathodic peak is more suppressed. The question is then why the intensity of CV peaks depends strongly on the ionic strength. A survey of literature studies reveals four potential roles played by the supporting electrolyte.

**Figure 2:**
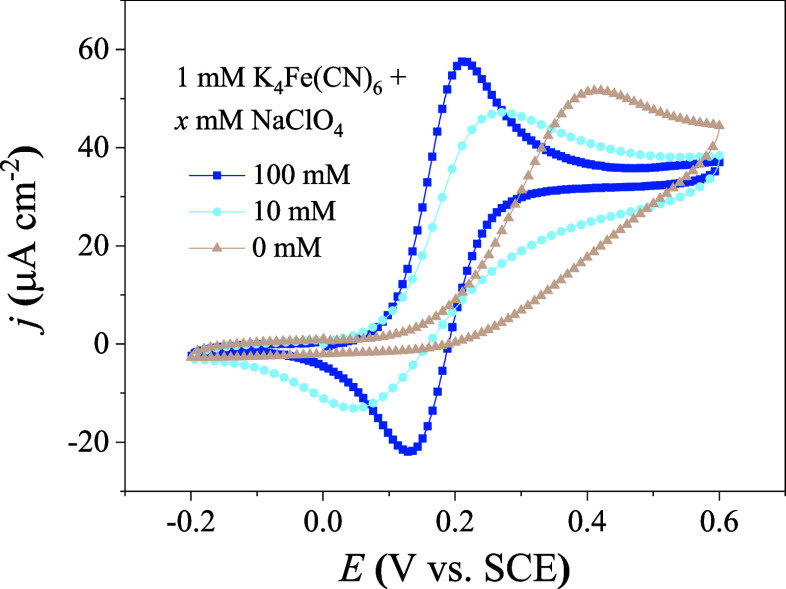
Influence of the concentration of the supporting electrolyte on CV profiles. CV curves are measured in a 1 mM K_4_Fe­(CN)_6_ aqueous solution with different concentrations of the supporting electrolyte (NaClO_4_). The scanning rate is *v* = 10 mV/s. The ohmic compensation ratio is 85%. All experimental data are recorded in the first cycle.

First, the most established role of supporting electrolytes is to influence the migration of reactive ions. Amatore *et al.* ^48^ and Copper *et al.* ^49^ derived an analytical expression to quantify the migration effects on the steady-state limiting current. The migration effects on the peak current in transient CV measurements have been investigated by Belding and Compton.^50^ Their findings consistently indicate that the contribution of migration is determined by both the charge and identity of the initial reactant. For example, when [Fe­(CN)_6_]^4–^ is used as the initial reactant, both the anodic and cathodic peaks should be enhanced in the absence of a supporting electrolyte, which is inconsistent with experiments exhibiting severely suppressed cathodic peaks. Therefore, the suppressed peak cannot be ascribed to migration effects only.

Second, the presence of a supporting electrolyte changes the ionic strength, thus modifying the structure of the EDL and altering the electron transfer kinetics at the reaction plane. Rooney *et al.* proposed that the like-charged [Fe­(CN)_6_]^3–^ ions are repelled when the electrode surface is negatively charged, resulting in a suppressed cathodic peak.^30^ The recent study of Levey *et al.* also suggested that the electrostatic repulsion between the electrode and the redox species reduces the peak current.^28^ However, it remains unclear why the anodic peak of [Fe­(CN)_6_]^4–^ is not equally suppressed despite the same repelling force of the EDL.

Third, decreasing the supporting electrolyte concentration increases the solution resistance, leading to a larger ohmic drop. A larger ohmic drop should reduce the effective driving force of the reaction, decreasing the current peak and increasing the peak separation. Since the ohmic drop should affect both anodic and cathodic peaks, the suppressed cathodic peak observed in Figure 2 cannot be attributed to ohmic drop alone. It is noted that ohmic compensation has been implemented in both experimental measurements and theoretical simulations, following previous studies.^28,50–52^

Fourth, the supporting electrolyte cations can directly interact with the reactive anions, altering their solvation shell and thus the solvent reorganization energy (λ) associated with electron transfer. The kinetic constants of [Fe­(CN)_6_]^3–/4–^ were found to depend on both the identity and concentration of cations.^44–46,53^ Campion *et al.* reported that the rate of isotopic exchange between [Fe­(CN)_6_]^3–^ and [Fe­(CN)_6_]^4–^ in aqueous solution increases at higher concentrations of cations.^54^ The variation of the kinetic constant is attributed to cation–anion association, which modifies the solvent reorganization energy. However, the influence of solvent reorganization energy is much less discussed in the context of EDL effects on CV.^23,28,55,56^ It is known that the difference between MHC kinetics—which explicitly includes λ—and EGV kinetics becomes more pronounced when λ is small.^37,38^ Therefore, it is essential to employ MHC kinetics, which captures variations in solvent reorganization induced by the changing ionic environment.

The four aforementioned roles are to be examined in the next section using the theoretical model.

### Parametric Analysis of Nonequilibrium EDL Effects on Peak Suppression

Numerical simulations were conducted in this section to analyze nonequilibrium EDL effects on the CV curves, focusing on peak suppression, using the one-variable-at-a-time method.

First, the ion migration effect was investigated by varying the concentration of the supporting electrolyte (*c*_sp_). Since changes in *c*_sp_ simultaneously modify the EDL structure, ion migration effects are examined for the special case of *E* ^0^ = *E*_pzc_ in which the EDL effects are minimized. As shown in Figure 3a, the cathodic and anodic peaks are equal when *c*_sp_ = 100 mM. The curve obtained by the full model almost overlaps with that of the diffusion model (blue circles). When *c*_sp_ = 0 mM, ohmic polarization and migration effects become significant. The former is eliminated by ohmic compensation (β = 100%), as detailed in Section S2 of the Supporting Information. After full compensation, the peak separation of the CV decreases to ∼60 mV, which is consistent with that of the diffusion model. As regards the migration effect, diffusion + migration models, such as the model of Mehandzhiyski et al.^57^ and Compton et al.,^50,58^ are included for comparison. The EDL structure and Frumkin corrections are neglected in diffusion + migration models. Compared to the case of *c*_sp_ = 100 mM, larger CV peaks are observed for the case of *c*_sp_ = 0 mM. In addition, the diffusion + migration model represented by gray circles neatly overlaps with the full model, proving that the EDL effect is minimal for the case of *E* ^0^ = *E*_pzc_. The increased peaks indicate that migration improves the transport of reactive ions. The simulation shows that migration effects impact both the anodic and cathodic peaks in a similar manner; thus, they are not responsible for the unequal peaks in CV curves.

**Figure 3:**
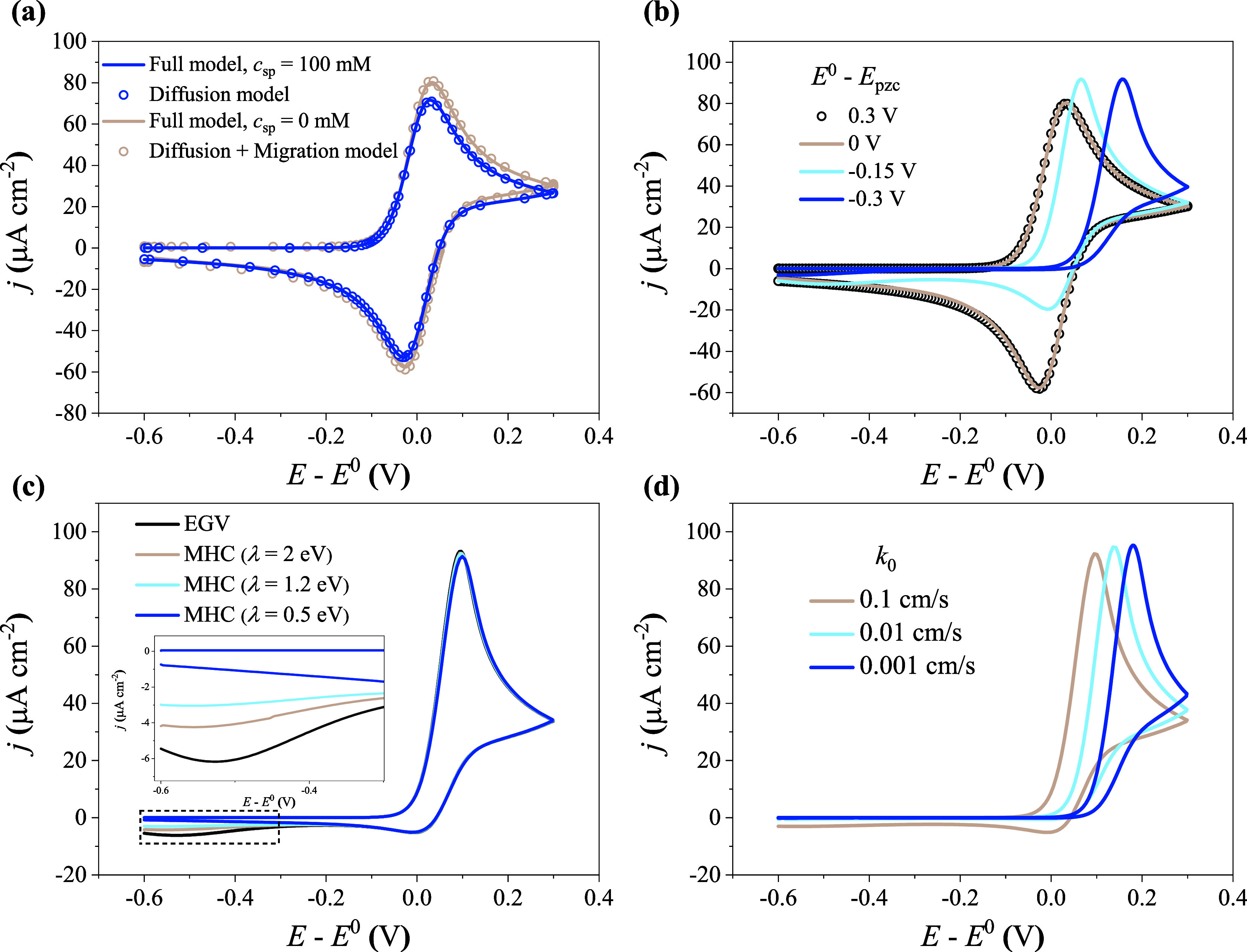
One-variable-at-a-time parametric analysis for nonequilibrium EDL effects on CV curves. (a) Comparison between the full model, the diffusion model, and the diffusion + migration model for the special case of *E* ^0^ = *E*_pzc_. The electrolyte solution is composed of 1 mM reactant (*z* = −4) + *x* mM supporting electrolyte (*c*_sp_). (b) The influence of PZC on the suppression of cathodic peaks in the absence of the supporting electrolyte, namely, *c*_sp_ = 0 mM. (c) The influence of the reorganization energy (*λ*) for the case of *c*_sp_ = 0 mM, *E* ^0^ – *E*_pzc_ = −0.2 V. (d) The influence of the kinetic constant (*k*_0_) for the case of *c*_sp_ = 0 mM, *E* ^0^ – *E*_pzc_ = −0.2 V. EVG kinetics is used in simulations (a) and (b), while MHC kinetics is used in simulations c and d. All the simulations are based on 1 mM K_4_Fe­(CN)_6_ solution, with a scanning rate of 10 mV/s and ohmic compensation ratio (β) of 100%.

Second, the PZC was varied to tune the EDL effects. The results reveal that CV is more sensitive to nonequilibrium EDL effects when the reactant is repelled from the electrode surface. As illustrated in Figure 3b, when *E* ^0^ – *E*_pzc_ shifts from 0 to −0.3 V, the anodic peak potential shifts positively and sharpens, while the original cathodic peak near −0.03 V is suppressed and finally disappears. Concurrently, a second cathodic peak at −0.5 V emerges and shifts negatively with a decreased *E* ^0^ – *E*_pzc_. The suppressed cathodic peak is attributed to the repelling force of the like-charged surface on reactive ions. The reactive species in the diffusion layer cannot be rapidly consumed. Once the electrode potential is sufficiently negative, the reduction of residual species in the diffusion layer results in a second cathodic peak. A similar phenomenon has been observed in the reduction of peroxydisulfate on steady-state voltammetry. The limiting current initially decreases due to the repulsion of the electrode to peroxydisulfate ions and rises again at a higher polarization potential.^7,17^ Interestingly, the anodic peak is not suppressed despite electrostatic repulsion, which will be discussed in the next section. However, when *E* ^0^ > *E*_pzc_, we do not observe any enhanced features in CV curves despite the attractive force of the EDL. The reason is that under fast kinetics conditions, the Nernstian equilibrium is nearly held.^28^ The current is completely controlled by mass transport. In summary, the CV curves exhibit suppressed peaks and greater sensitivity to nonequilibrium EDL effects when the reactant is repelled from the electrode surface.

While the model predicts a larger anodic peak in the absence of a supporting electrolyte, namely, when the nonequilibrium EDL effects become prominent, experiments show smaller anodic peaks (Figure 2). This discrepancy could be attributed to the concentration-dependent diffusion coefficient,^59,60^ natural convection,^61,62^ and EDL effects other than the Frumkin correction, such as solvent orientational polarization,^63,64^ specific adsorption,^65^ and partial ion desolvation.^66,67^

Third, we evaluated the role of solvent reorganization energy (λ) in the EDL effects. A lower λ facilitates the suppression of peaks at high overpotentials, thereby amplifying the visibility of nonequilibrium EDL effects. As shown in Figure 3c, the anodic peak and the first cathodic peak are almost independent of λ, as the kinetic rate constants predicted by the MHC and EGV models differ only marginally at low overpotentials. The significant difference between the two types of kinetics in CV could be amplified by high scanning rates and slow electron transfer.^38^ In contrast, the second cathodic peak, which appears at a high overpotential, is highly sensitive to λ. As λ decreases, the second cathodic peak deviates from the prediction of the EGV kinetics and eventually disappears, demonstrating the distinct role of solvent reorganization under large driving forces. λ = 1.2 eV has been reported for [Fe­(CN)_6_]^3–/4–^,^47^ and the corresponding second cathodic peak is invisible. The comparison of MHC and EGV theories shows that the peak suppression by nonequilibrium EDL effects is generic to different theories of electron transfer. A lower λ amplifies the peak suppression induced by nonequilibrium EDL effects at a high overpotential.

Fourth, a lower kinetic constant (*k*_0_) enhances the peak suppression induced by nonequilibrium EDL effects. Several studies have reported that the concentration of the counter cation, such as K^+^, alters the *k*_0_ of the [Fe­(CN)_6_]^3‑/4‑^ redox reaction.^44,46^ As shown in Figure 3d, when *k*_0_ decreases from 0.1 to 0.001 cm/s, the anodic peak obtained by the full model shifts positively, while the cathodic peak almost disappears. To isolate the effect of *k*_0_, we examined the impact of decreased *k*_0_ using the diffusion model without considering EDL effects, as demonstrated in Section S3 of the Supporting Information. The diffusion model shows that the anodic peak shifts positively as *k*_0_ decreases, while the cathodic peak remains clearly present, indicating that the suppression of the cathodic peak in the experiment cannot be fully attributed to a lower *k*_0_.

The parametric analysis indicates that the suppressed peaks in CV profiles are dominated by nonequilibrium EDL effects. Specifically, the cathodic peak is suppressed when the reactant is repelled electrostatically from the electrode surface, as modulated by *E* ^0^ – *E*_pzc_. The model also suggests that lower λ and *k*_0_ values should enhance the peak suppression.

### Model-Based Spatiotemporal Distributions of Electrostatic Potential and Concentration

The peaks of CV curves are convoluted in that the peak results from the accumulation of all previous steps. Hence, it is often insufficient to discuss nonequilibrium EDL effects only at the point of the peak. Instead, the whole spatiotemporal distribution of electrostatic potential and concentration must be considered. One aspect of the challenges is to estimate the magnitude of the current contributed by the concentration gradient inside the EDL region within six Debye lengths (6λ_D_) from the electrode surface due to the overwhelming influence of local electrostatic interactions. In contrast, the concentration profiles in the outer part of the EDL are mainly controlled by mass transport rather than the mean-field electrostatic force. Therefore, the region from 6 to 10λ_D_ away from the electrode surface is defined as a transition region between the EDL and the diffusion layer. This region is key to understanding nonequilibrium EDL effects on the CV peaks.

Figure 4 shows the electrostatic potential and concentration distributions in the region within 6λ_D_ of the electrode surface as a function of the applied potential. The spatial coordinates are divided into two parts, including the EDL (0 < *x* < 6λ_D_) and the transition region (6λ_D_ < *x* < 10λ_D_) between the EDL and the diffusion layer. Reactive ions [Fe­(CN)_6_]^4–^ have a negative valence. The electrode potential (*E*) sweeps from the lower limit (*E*_1_ = −0.6 V) to the upper limit (*E*_2_ = 0.3 V) for oxidation and back from *E*_2_ to *E*_1_ for reduction.

**Figure 4:**
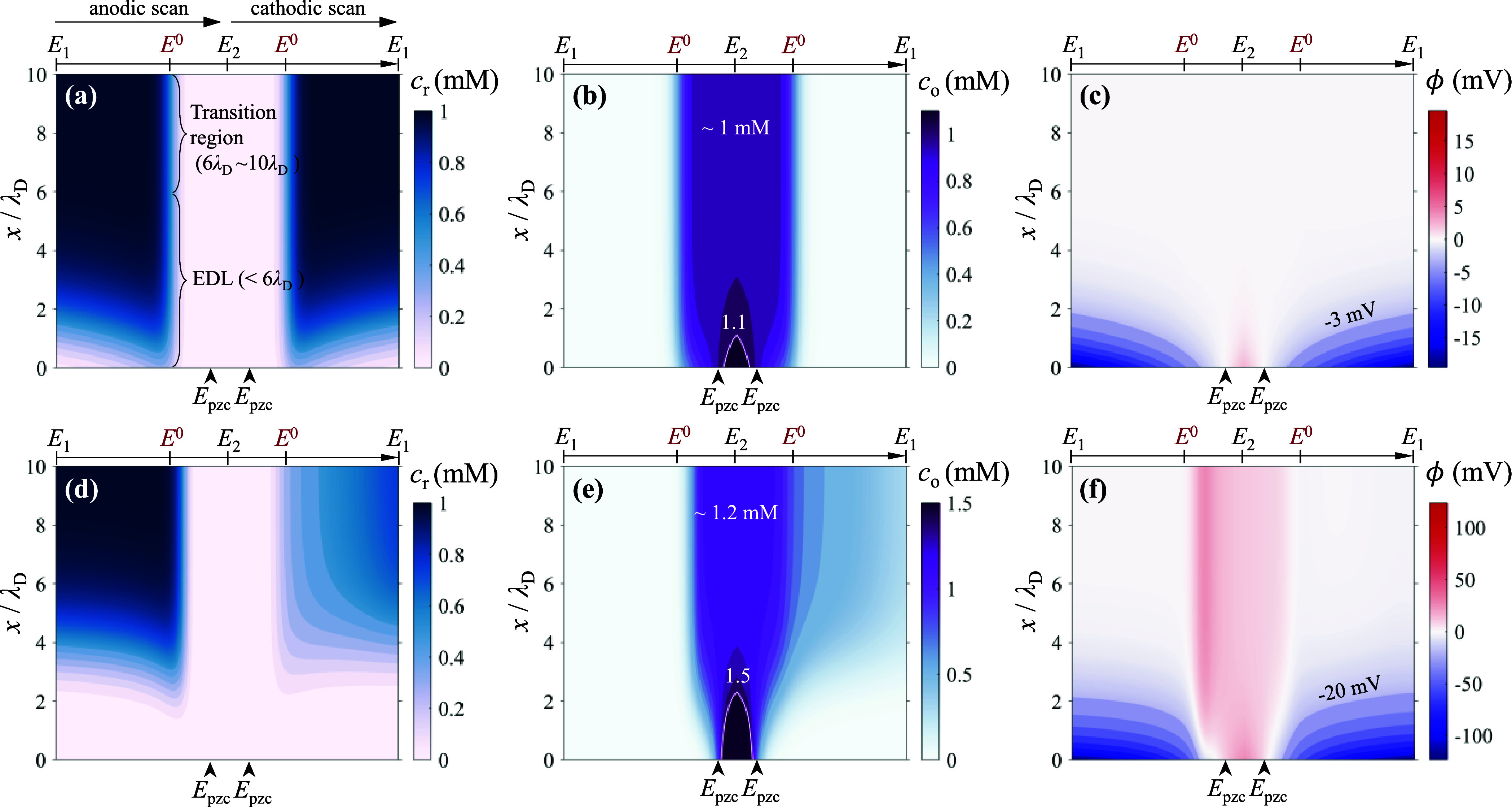
Electrostatic potential and concentration distributions as a function of the electrode potential: (a, d) reductant concentration, *c*_r_; (b, e) oxidant concentration, *c*_0_; (c, f) electrostatic potential, ϕ. Top panels correspond to 1 mM reactant (*z* = −4) with 1000 mM supporting electrolyte, while the bottom panels correspond to the case without a supporting electrolyte. The spatial coordinate *x* is normalized by the Debye length, λ_D_. A typical value of *E* ^0^ – *E*_pzc_ = −0.2 V is used to discuss nonequilibrium EDL effects. The scanning rate is 10 mV/s. Other parameters are the same as those in Table 1. To highlight concentration variations in the transition region (6λ_D_ < *x* < 10λ_D_) between the EDL and the diffusion layer, concentrations exceeding the white contour line are plotted in the same color.

Figure 4a,b present the concentration distributions of the reductant (*c*_r_) and oxidant (*c*_0_) in the presence of an abundant supporting electrolyte (*c*_sp_ = 1000 mM). It is shown that the changes in the concentration and electrostatic potential within the EDL have minimal influence on CV in this case. During *E* sweeps from *E*_1_ to *E*_2_, the oxidation commences at *E* ^0^. As a result, *c*_r_ decreases from 1 to 0 mM, while *c*_0_ increases from 0 to 1 mM in the transition region, corresponding to the anodic peak in the CV curves. In contrast, concentrations in the EDL are mainly governed by the local electrostatic potential rather than mass transport. As is evident in Figure 4c, when *E* exceeds *E*_pzc_, the interfacial electrostatic potential transitions from negative to positive values. Consequently, *c*_r_ and *c*_0_ in the EDL vary synchronously with the electrostatic potential therein. Commonly, *c*_r_ and *c*_0_ are lower than 1 mM when *E* < *E*_pzc_, and they become higher than 1 mM when *E* > *E*_pzc_. Since the reductant has been consumed in the reaction, *c*_r_ in the EDL remains below 1 mM when *E* > *E*_pzc_. The concentrations and potential at the HP have a minimal effect on the CV response in the presence of a sufficient supporting electrolyte, which is rationalized by the fact that ϕ_H_ is nearly zero.

By comparison, without the shielding effect of supporting ions (*c*_sp_ = 0 mM), the concentrations and electrostatic potentials at the HP determine the CV profile. As shown in Figure 4f, the electrostatic potential in the EDL is more negative than that in the presence of supporting ions. As a result, the more negative potential at the HP reduces the local redox species concentration on the one hand and enhances the effective overpotential for oxidation on the other. The enhancement in overpotential compensates for the reduced reactant concentration, which explains why the anodic peak of [Fe­(CN)_6_]^4–^ is not suppressed despite the repelling force of the EDL toward [Fe­(CN)_6_]^4–^.

During the reverse potential sweep from *E*_2_ to *E*_1_, the suppression of the cathodic peak is a result of the collective effect of the depleted reactant concentration and diminished effective overpotential. In the presence of a supporting electrolyte, *c*_r_ (Figure 4a) and *c*_0_ (Figure 4b) in the transition region return to their bulk concentrations. The corresponding CV curve exhibits a cathodic peak with a height comparable to that of the anodic peak, indicating that the EDL has minimal impact on the CV shape in this case. However, the EDL strongly suppresses the cathodic peak in the absence of a supporting electrolyte. As mentioned above, a low ionic strength leads to both reduced concentration and more negative ϕ_H_ when *E* < *E*_pzc_. The latter results in a lower effective overpotential for the reduction reaction. These two effects collectively suppress the cathodic peak. Therefore, as shown in Figure 4d,e, *c*_0_ in the transition region is not completely consumed when *E* falls in the range from *E* ^0^ to *E*_1_, while *c*_r_ does not return to its initial concentration of 1 mM.

In addition, the distribution of *c*_0_ in the transition region also reveals the impact of ion migration on the diffusion layer in the absence of a supporting electrolyte. The poor conductivity leads to a noticeable electrostatic potential gradient across the solution phase, driving ion migration. The migration effect alters the mass transport rate of the ions and consequently the reaction current.^48–50^ According to the IUPAC convention for current direction,^68^ the Nernst–Planck equation is expressed as

$$\frac{j_{\mathrm{ct}}}{F}=\pm D\frac{\partial c}{\partial x}\pm \frac{zDF}{RT}c\frac{\partial \phi }{\partial x}$$ 20

the signs “+” and “–” correspond to the reductant and oxidant, respectively. The second term on the right-hand side of the equation represents the contribution of migration to the current, which depends on the reactant charge (*z*), the sign of the potential gradient, and the identity of the initial reactive species. Once *E* exceeds *E* ^0^, the potential gradient in the transition region becomes negative (Figure 4f). When the reductant (*z* < 0) is the initial reactant, the resulting negative potential gradient drives the reactive anion toward the electrode. Therefore, we find that *c*_0_ in the transition region tends to reach a plateau at around 1.2 mM due to enhanced migration (Figure 4e), which is higher than the value observed in cases with a sufficient supporting electrolyte. The increased concentration results in an increased *j*_ct_, thereby enhancing the total peak current.

In summary, the suppression of the cathodic peak is attributed to two interfacial changes induced by EDL effects, namely, decreased reactant concentrations and decreased effective overpotentials for reduction. In contrast, during oxidation, the impact of decreased concentrations is compensated for by an increased overpotential, retaining the anodic peak. This unequal modulation of the concentration and driving force by the EDL is the key mechanism behind the unequal CV peaks. In addition, the migration effect directly increases the concentration in the transition region, increasing the peak current.

### A Regime Diagram of Nonequilibrium EDL Effects in CV

Based on the above analysis, we understand that the CV curves are mainly governed by two factors. The first factor is the migration effect on mass transport, which depends on the reactant charge (*z*) and the identity of the initial reactive species. The second factor is the EDL effect on the reaction kinetics, which is determined by the charge of both the reactant and the electrode surface. The electrode surface charge is determined by the formal potential with respect to PZC, *E* ^0^ – *E*_pzc_. To examine these effects, we perform a parametric analysis for the CV curves without the supporting electrolyte (black solid lines) in the parametric space of *z* and *E* ^0^ – *E*_pzc_, with the case of a sufficient supporting electrolyte serving as a comparison (gray dashed lines). Figure 5a,b show two separate cases based on the initial species, with the prefixes “R” and “O” denoting the reductant and oxidant, respectively. The identity of the initial species determines the initial direction of potential scanning: oxidation starts in Figure 5a, whereas reduction starts in Figure 5b. The CV profiles in each figure are divided into four cases according to the values of two parameters: *z* and *E* ^0^ – *E*_pzc_.

**Figure 5:**
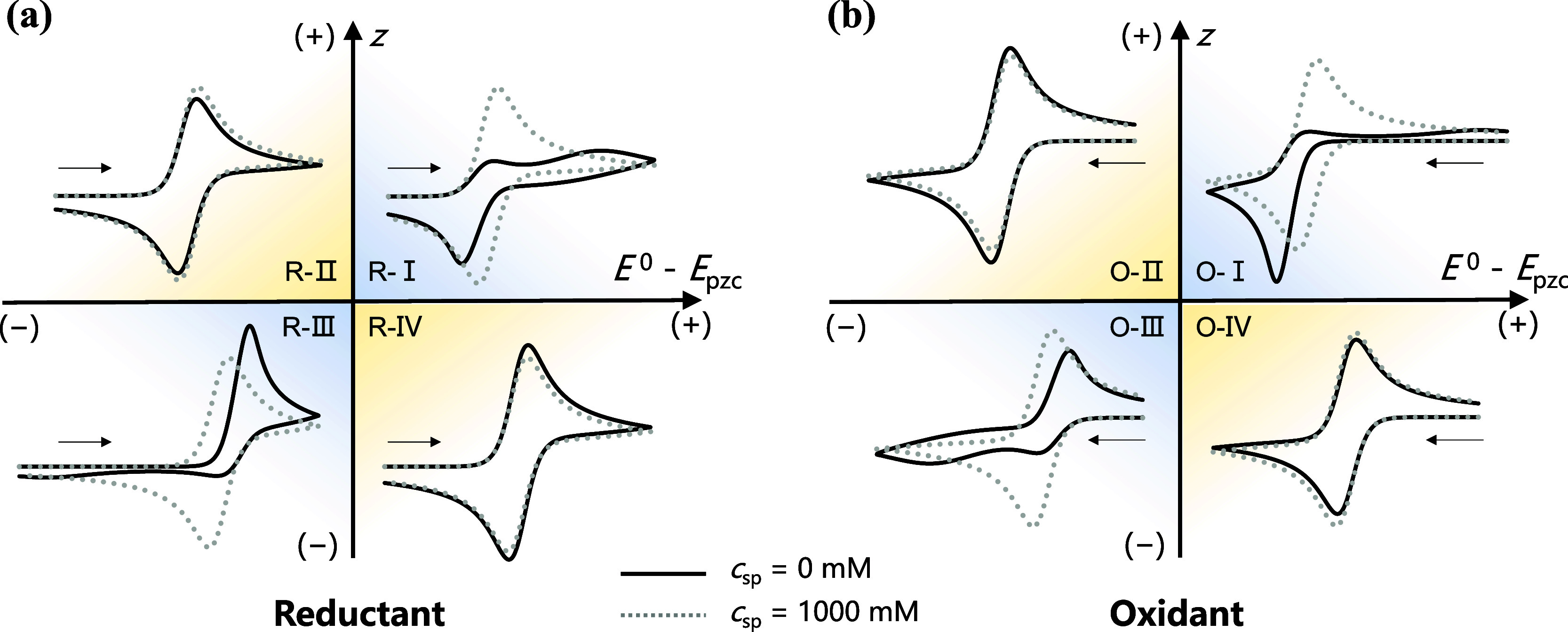
Regime diagram of nonequilibrium EDL effects as a function of the reactant charge (*z*) and the formal potential with respect to the PZC, *E* ^0^ – *E*_pzc_. Black solid lines represent CV profiles of 1 mM reactant in the absence of the supporting salt, while dashed lines represent the cases with the 1000 mM supporting electrolyte. The initial reactants are reductant (a) and oxidant (b). The cases I, II, III, and IV are classified based on the charges of the reactants and electrode; the prefixes “R” and “O” refer to reductants and oxidants as initial reactants, respectively. The kinetic constant is 0.1 cm/s. The scanning rate is 10 mV/s. The compensation ratio is 100%.

The oxidation of K_4_Fe­(CN)_6_ discussed earlier belongs to case R-III in Figure 5a, where the repulsion of the EDL suppresses the cathodic peak. There is an analogous case, i.e., case R-I, *z* > 0 and *E* ^0^ – *E*_pzc_ > 0, in which the anodic peak is suppressed. As illustrated in the electrostatic potential distribution for case R-I in Figure S5a, the electrode surface is positively charged when the applied potential *E* is around *E* ^0^, repelling reactive cations and decreasing the surface concentrations *c*_r_ and *c*_0_ (see Figures S6a and S7a in Section S4 of the Supporting Information). Moreover, the positive potential at the HP reduces the effective overpotential for oxidation. The synergy between decreased concentrations and reduced effective overpotential collectively suppresses the oxidation kinetics. Like case R-III, a second anodic peak was also observed at higher overpotential in case R-I. As shown in Figure 5b, when the initial reactant is the oxidant, the EDL effects on the dependence of *z* and *E* ^0^ – *E*_pzc_ are consistent with those shown in Figure 5a. Specifically, the anodic peak is suppressed in the case of O-I, while the cathodic peak is suppressed in the case of O-III. In other words, the repelling force of the electrode toward reactive ions is responsible for suppressing the reduction of anions (cases R-III and O-III) and the oxidation of cations (cases R-I and O-I), resulting in suppressed peaks in CV curves.

In contrast to the repulsive cases, the EDL exerts an attractive interaction on the reactive ions in cases R-II, R-IV, O-II, and O-IV, which theoretically enhances the reaction kinetics. Levey *et al*. reported that the attraction of ions from the EDL can enhance the peak current and decrease peak separation under a kinetic regime of *k*_0_ = 0.01 cm/s and *v* = 1000 mV/s.^28^ However, as shown in cases R-II, R-IV, O-II, and O-IV, the influence of enhanced kinetics on the CV shape is less pronounced when *k*_0_ = 0.1 cm/s and *v* = 10 mV/s because the Nernstian equilibrium is nearly held and the current is primarily controlled by mass transport under the fast kinetics condition. Consequently, the effect of the EDL attraction on the CV profile is minimal. As a result, the observed peak variations are mainly caused by ion migration. The cases R-II, R-IV, O-II, and O-IV demonstrate that the contribution of migration to the current depends on the charge and identity of the initial reactant. Specifically, the ion migration is expected to decrease the current for the oxidation of cations or the reduction of anions and to increase that for the reduction of cations or the oxidation of anions. The results are consistent with the previous literature.^48–50^

In general, the suppression of the peak caused by the repelling force of the EDL depends on the reactant charge. While EDL attraction could theoretically enhance the kinetics and consequently the reversibility of the CV shape, the parametric space where this enhancement can be observed is much smaller. In other words, CV is more sensitive to nonequilibrium EDL effects when the reactant is repelled from the electrode surface than under attractive conditions.

### Experimental Verification of the Regime Diagram of the EDL Effects

The model-based regime diagrams of EDL effects are verified by measuring the CV responses of redox couples with different charges and kinetics.

As mentioned above, the CV response falls into case R-III when [Fe­(CN)_6_]^4–^ is the initial reactant. According to the classification in Figure 5, the CV response could correspond to case O-III when [Fe­(CN)_6_]^3–^ serves as the initial species, which is indeed supported by experimental results. As illustrated in Figure 6a, the cathodic peak is broader than the anodic peak in the absence of NaClO_4_. The cathodic peaks have lower magnitudes compared with the anodic peaks regardless of whether the initial reactant is [Fe­(CN)_6_]^4–^ or [Fe­(CN)_6_]^3–^. In other words, the nonequilibrium EDL effects are independent of the identity of the initial reactant. On the contrary, an alternative explanation for unequal peaks in CV, namely, sluggish redox kinetics, depends on the identity of the initial reactant. If nonequilibrium EDL effects were not considered, the anodic peak of quasi-reversible CV curves would be higher than the cathodic peak when the reductant serves as the initial reactant, whereas the opposite occurs when the oxidant is the initial reactant. This comparison convincingly shows that the suppressed CV peaks are due to nonequilibrium EDL effects rather than sluggish redox kinetics.

**Figure 6:**
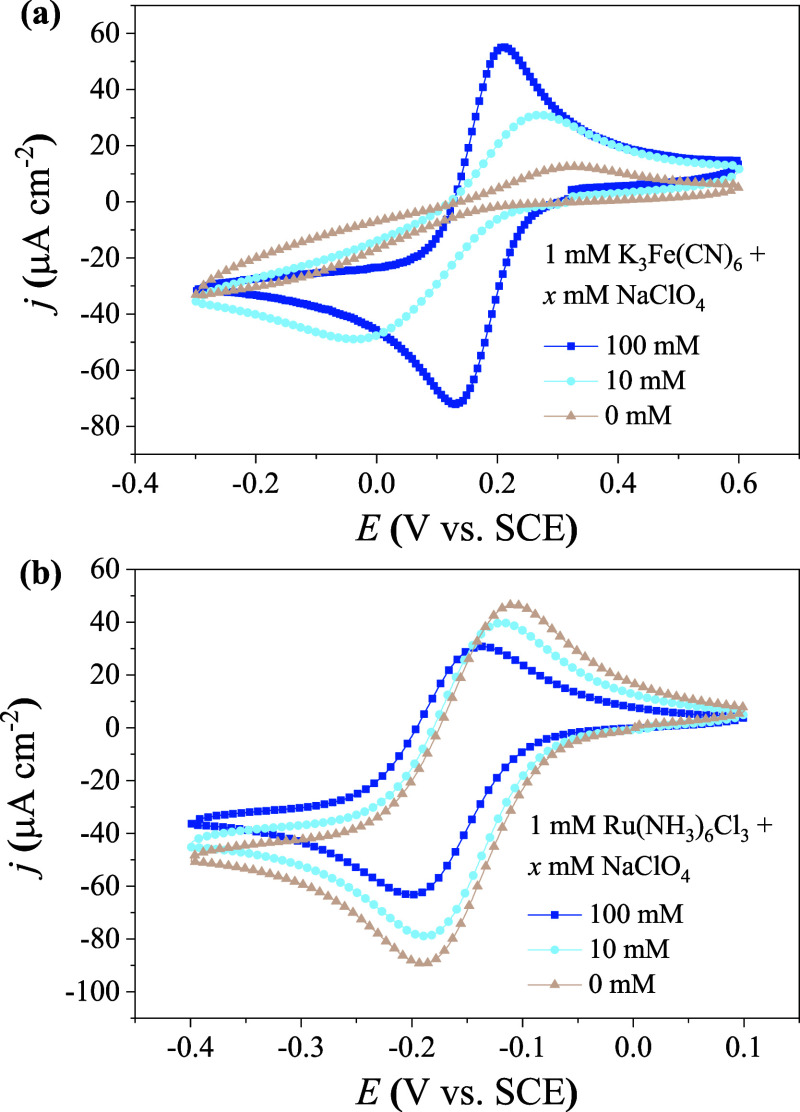
Comparison of CV profiles with varying concentrations of the supporting electrolyte for different redox couples of 1 mM: (a) K_3_[Fe­(CN)_6_] and (b) [Ru­(NH_3_)_6_]­Cl_3_. The ohmic compensation ratio is 85%. The scanning rate is 10 mV/s.

Compared to the reported kinetics of [Fe­(CN)_6_]^3–/4–^ (*k*_0_ = 0.001–0.1 cm/s),^42–46^ the kinetics of [Ru­(NH_3_)_6_]^3+/2+^ is much faster (*k*_0_ = 8–14 cm/s).^69,70^ As a result, the CV of [Ru­(NH_3_)_6_]^3+/2+^ is less perturbed by nonequilibrium EDL effects and remains reversible regardless of the ionic strength. As shown in Figure 6b, the CV curves of [Ru­(NH_3_)_6_]^3+/2+^ exhibit nearly equal peaks even in the absence of NaClO_4_. This comparison confirms that the severe suppression of CV peaks originates from the combination of slower redox kinetics and repulsive EDL effects.

Beyond reversibility, the variation in the CV peak heights of [Ru­(NH_3_)_6_]^3+/2+^ reveals an additional effect of the ionic strength. The peak intensities decreases by ∼40% as the concentration of NaClO_4_ increases. However, the migration effect accounts for only about 10% of the variation. The remaining variation is likely related to changes in the diffusion coefficient of the redox species. A similar phenomenon has been observed in other supporting salts, such as KCl, KNO_3_, and K_2_SO_4_, by Wang et al.^59^ Some studies have suggested that reactive ions can bind with supporting anions, such as sulfate^59^ and nucleotide phosphate,^60^ thereby altering the diffusion coefficient.

### Regulating Nonequilibrium EDL Effects via Changing the Scanning Rate (*v*)

The preceding analysis reveals that nonequilibrium EDL effects are modulated by the kinetic regime of the redox reaction. Experimentally, the scanning rate (*v*) serves as a key parameter controlling the effective kinetics of the system. By varying *v*, we can tune the system from a reversible state (slow scanning rates) to an irreversible state (fast scanning rates), thereby mapping out how nonequilibrium EDL effects evolve with effective kinetic conditions.

As illustrated in Figure 7a, in the weakly supported electrolyte (1 mM K_4_Fe­(CN)_6_ + 10 mM NaClO_4_ in aqueous solution), the cathodic peaks are gradually suppressed as the scanning rate increases from 3 to 500 mV/s. The ratios of cathodic to anodic peak currents (*j*_p,c_/*j*_p,a_) are used to quantify the degree of suppression of the cathodic peaks. It should be noted that the current values of the peaks are corrected with respect to the baselines; see Section S5 of the Supporting Information. As shown in Table S1, the ratio *j*_p,c_/*j*_p,a_ reduces with increasing scanning rate, indicating enhanced nonequilibrium EDL effects at higher scanning rates. Above a scanning rate of 10 mV/s, the cathodic peak significantly broadens and shifts negatively as the scanning rate increases. However, an increased scanning rate also induces irreversible CV profiles, resulting in unequal peak heights. This concern is addressed in Figure 7b. When K_3_Fe­(CN)_6_ is used as the initial reactive species, the ratio *j*_p,c_/*j*_p,a_ decreases as the scanning rate increases but remains less than 1 (see Figure S9 and Table S2). If the irreversibility of the CV curves was caused by the higher scanning rate, the initial cathodic peak should be greater than the subsequent anodic peak, corresponding to *j*_p,c_/*j*_p,a_ > 1. Therefore, *j*_p,c_/*j*_p,a_ < 1 in K_3_Fe­(CN)_6_ confirms that the suppressed cathodic peak is due to the nonequilibrium EDL effects. The systematic decrease in *j*_p,c_/*j*_p,a_ with *v* demonstrates that the nonequilibrium EDL effects are modulated by the scanning rate.

**Figure 7:**
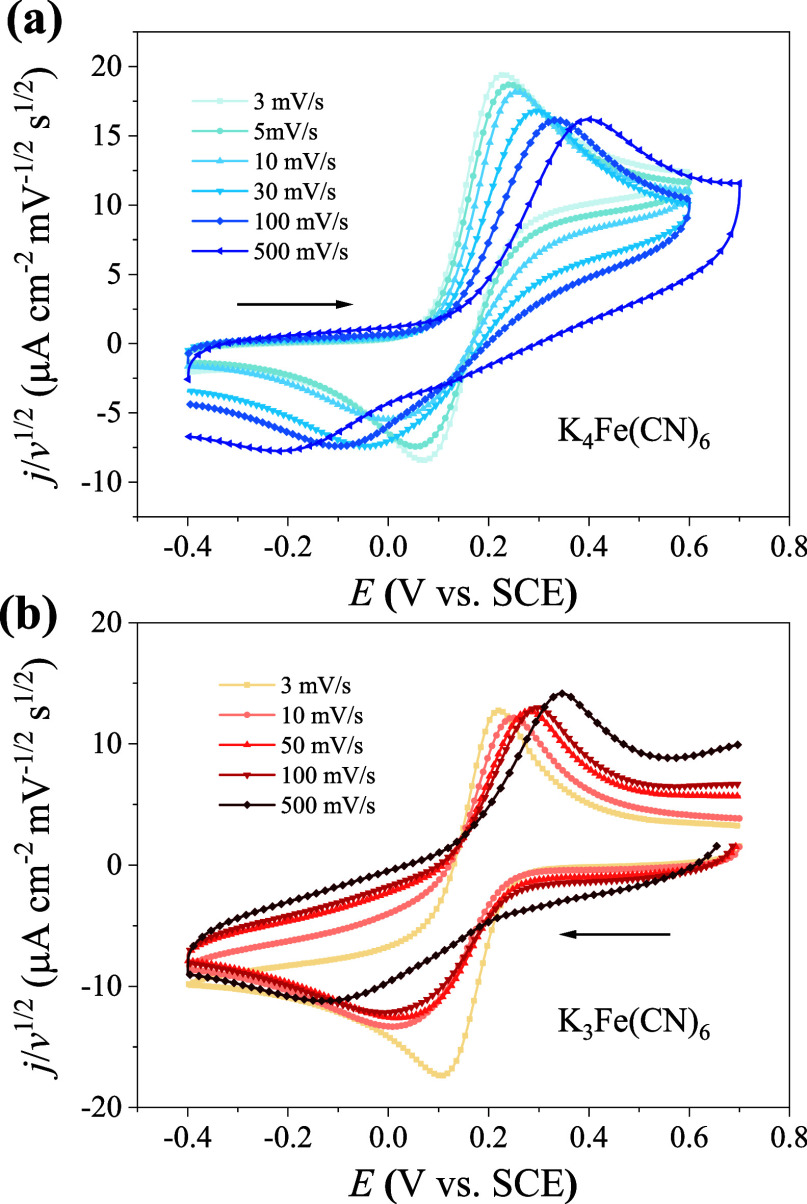
Nonequilibrium EDL effects are modulated by the scanning rates. The CV curves at different scanning rates in aqueous solution with 1 mM reactant + 10 mM NaClO_4_ for (a) K_4_Fe­(CN)_6_ and (b) K_3_Fe­(CN)_6_. The current densities are normalized by the square root of the scanning rate. The arrow points to the initial scanning direction.

Figure 8a,b present the simulated cathodic-to-anodic peak current ratio (*j*_p,c_/*j*_p,a_) as a function of ionic strength, dimensionless rate constant (*K*), and difference between the formal potential and the potential of zero charge (*E* ^0^ – *E*_pzc_). *K* is defined as^71^

$$K=\frac{k_{0}}{\sqrt{D\frac{F}{RT}v}}$$ 21

**Figure 8:**
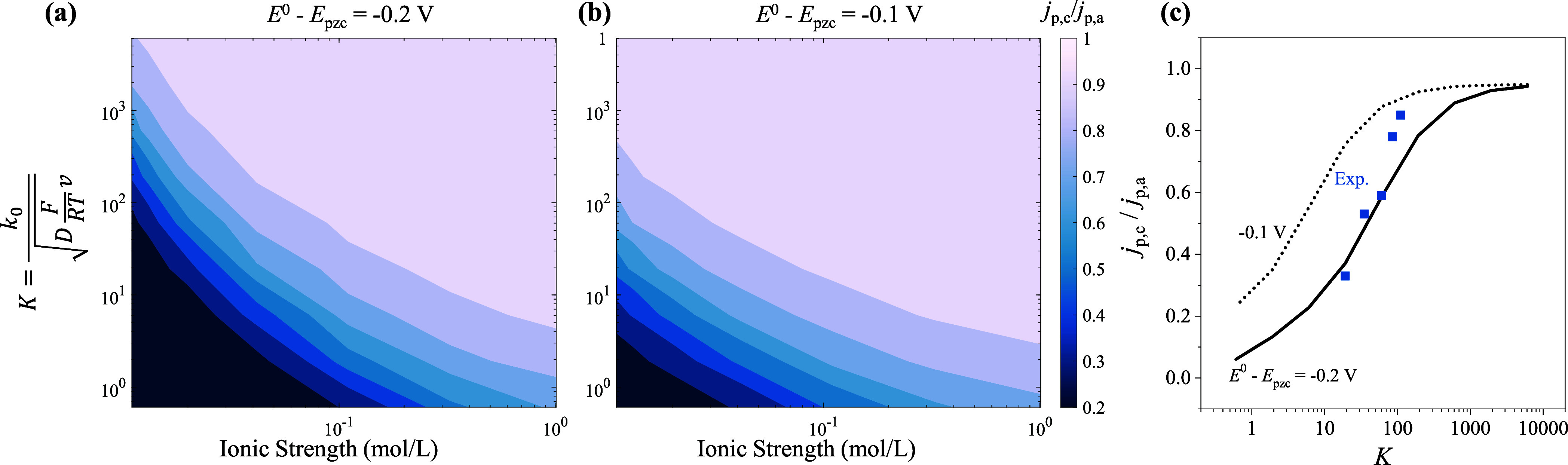
Simulated cathodic-to-anodic peak current ratio (*j*_p,c_/*j*_p,a_) as a function of ionic strength ( $\frac{1}{2}\underset{i}{\sum }c_{i}z_{i}^{2}$ ), dimensionless rate constant (*K*), and potential of zero charge (*E* ^0^ – *E*_pzc_). The regimes (a) and (b) are obtained at *E* ^0^ – *E*_pzc_ = −0.2 and −0.1 V, respectively. The charge of the initial reactive species is −4. (c) Experimental *j*_p,c_/*j*_p,a_–*K* data (blue dots) are calculated from Figure 7a, with *k*_0_ = 0.1 cm/s. The solid and dashed lines represent the *j*_p,c_/*j*_p,a_–*K* curves at *E* ^0^ – *E*_pzc_ = −0.2 and −0.1 V, respectively.

As the ionic strength decreases, *j*_p,c_/*j*_p,a_ decreases monotonically, indicating suppression of the cathodic peak with respect to the anodic peak. This distortion of the CV shape becomes more pronounced for smaller *K* values, confirming that EDL effects are amplified when the electron transfer is less reversible. Under low-ionic-strength conditions, the shift of *E* ^0^ – *E*_pzc_ from −0.2 to −0.1 V lowers the threshold *K* value for the onset of suppression. These diagrams quantitatively establish a direct relationship between the parameters of the system and the degree of the CV distortion observed experimentally. Figure 8c presents the peak ratio calculated from Figure 7a. The solid and dashed lines represent the *j*_p,c_/*j*_p,a_–*K* curves at *E* ^0^ – *E*_pzc_ = −0.2 and −0.1 V, respectively, both at an ionic strength of 0.02 mol/L. The experimental peak ratios decrease with decreasing *K* and fall between the two working curves. This comparison is not satisfactory, as it is challenging to determine all of the model parameters. Our simplified model does not fully capture the complexities of the real system. Nevertheless, Figure 8a,b can serve as a valuable guide for experimentalists to estimate the dominant EDL regime under their specific conditions.

## Conclusions

CV profiles with unequal peaks were experimentally observed. A comparative analysis of four possible causes leads us to conclude that the unequal CV peaks are attributed mainly to nonequilibrium EDL effects. In one scanning direction, the CV peak is suppressed synergistically by a decrease in the reactant concentration and a decrease in the effective overpotential in the EDL. On the contrary, in the opposite scanning direction, the impact of decreased concentrations is compensated for by an increased overpotential, diminishing the suppression effect of the EDL. A regime diagram has been obtained using the theoretical model to characterize nonequilibrium EDL effects on CV curves in terms of two key variables: reactant charge (*z*) and the formal potential with respect to PZC (*E* ^0^ – *E*_pzc_). This diagram is divided into two regimes depending on the electrostatic force exerted by the EDL on the reacting ion, either repelling or attractive. The repelling force of the EDL can result in the unilateral suppression of the CV peak, while the attractive force usually does not unless at ultralow electrode kinetics. Finally, nonequilibrium EDL effects are more pronounced at a higher scanning rate.

## Supplementary Material



## Notes

Conversion of electrode potentials and electric potentials, ohmic polarization and ohmic compensation, influence of the kinetic constant on the CV curves in the pure diffusion model, spatiotemporal distributions of the electrostatic potential and the concentration, ratios of cathodic to anodic peaks and peak separations, and step-by-step tutorial of the implementation of the model in COMSOL (PDF)
