# Supplementary material for: Nonequilibrium Electrical Double Layer Effects on Unilateral Peak Suppression in Cyclic Voltammetry
Source: ACS Electrochem. 2026 Apr 10;2(5):1148–60. doi: 10.1021/acselectrochem.5c00491 (PMC13158924; doi:10.1021/acselectrochem.5c00491)
Supplement: Supplementary file 1 [file ec5c00491_si_001.pdf]

Supporting Information for

Nonequilibrium Electrical Double Layer Effects on Unilateral Peak Suppression in Cyclic Voltammetry

Yupeng Qin[1,2], Zhangquan Peng[1,*], Jun Huang[3,4,*]

[1] *Laboratory of Advanced Spectro-electrochemistry and Li-ion Batteries, Dalian Institute of Chemical Physics, Chinese Academy of Sciences, Dalian 116023, China*

[2] *University of the Chinese Academy of Sciences, Beijing 100049, China*

[3] *Institute of Energy Technologies, IET-3: Theory and Computation of Energy Materials, Forschungszentrum Jülich GmbH, Jülich 52425, Germany*

[4] *Faculty of Georesources and Materials Engineering, RWTH Aachen University, Aachen 52062, Germany*

* Corresponding author: zqpeng@dicp.ac.cn; ju.huang@fz-juelich.de

Table of Contents

S1: Conversion between model parameter (ϕM $-$ χM) and experimental variables (E $-$ Epzc) 2

S2: Ohmic polarization and ohmic compensation 4

S3: The influence of the kinetic constant on the CV curves in the pure diffusion model 6

S4: Spatiotemporal distributions of the electrostatic potential and the concentration 7

S5: The ratios of cathodic to anodic peaks and peak separations 12

S6: Step-by-step tutorial of implementation of the model in COMSOL 14

S1: Conversion between model parameter ($\phi_M - \chi_M$) and experimental variables ($E - E_{pzc}$)

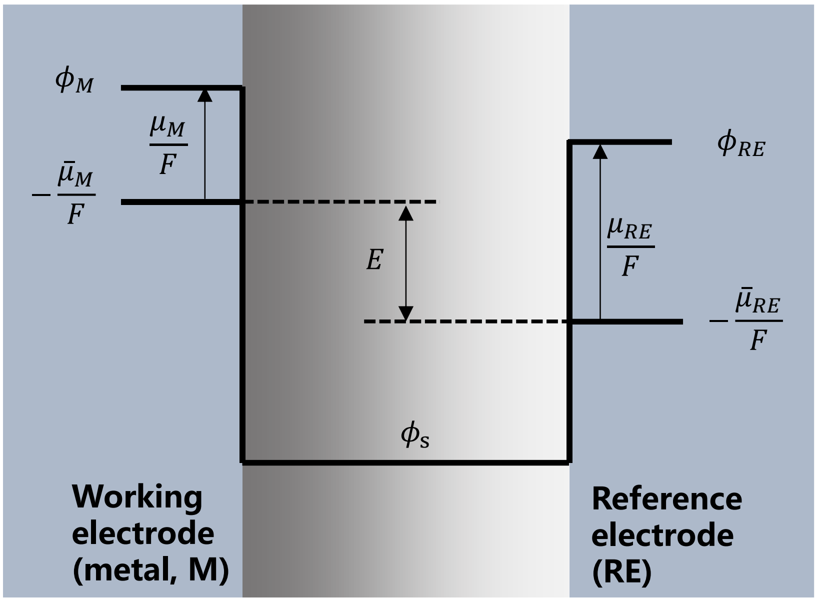

Figure S1. The interfacial potential difference is controlled by the electrode potential, which is defined as the difference in the electrochemical potential of electrons between the metal electrode ($\bar{\mu}_M$) and the reference electrode ($\bar{\mu}_{RE}$).

The relation between the inner potential (ϕ_i) and the electrochemical potential ($\bar{\mu}_i$) is

$$\phi_i = \frac{\mu_i}{F} - \frac{\bar{\mu}_i}{F}, \tag{S1}$$

where μ_i is the chemical potential of the electrons in electrode i.

Electrode potential (E) is expressed by the difference of the electrochemical potential of the electron of the metal ($\bar{\mu}_M$) and the reference electrode ($\bar{\mu}_{RE}$)

$$E = -\frac{\bar{\mu}_M - \bar{\mu}_{RE}}{F} = \phi_M - \phi_{RE} - \frac{\mu_M - \mu_{RE}}{F} = (\phi_M - \phi_s) - (\phi_{RE} - \phi_s) - \frac{\mu_M - \mu_{RE}}{F}. \tag{S2}$$

Therefore, the interfacial potential difference is given as,

$$\phi_M - \phi_s = E + (\phi_{RE} - \phi_s) + \frac{\mu_M - \mu_{RE}}{F}. \tag{S3}$$

Subtracting χ_M from both sides of the equation leads to,

$$\phi_M - \phi_s - \chi_M = E + (\phi_{RE} - \phi_s) + \frac{\mu_M - \mu_{RE}}{F} - \chi_M. \tag{S4}$$

When the electrode potential is exactly at the potential of zero charge, we get,

$$\phi_M - \phi_s = \chi_M, \tag{S5}$$

and

$$E_{\mathrm{pzc}} = \chi_M - (\phi_{RE} - \phi_s) - \frac{\mu_M - \mu_{RE}}{F}. \tag{S6}$$

As we define $\phi_s = 0$, eq S4 is rewritten as

$$\phi_{\mathrm{M}} - \chi_{\mathrm{M}} = E - E_{\mathrm{pzc}}. \tag{S7}$$

S2: Ohmic polarization and ohmic compensation

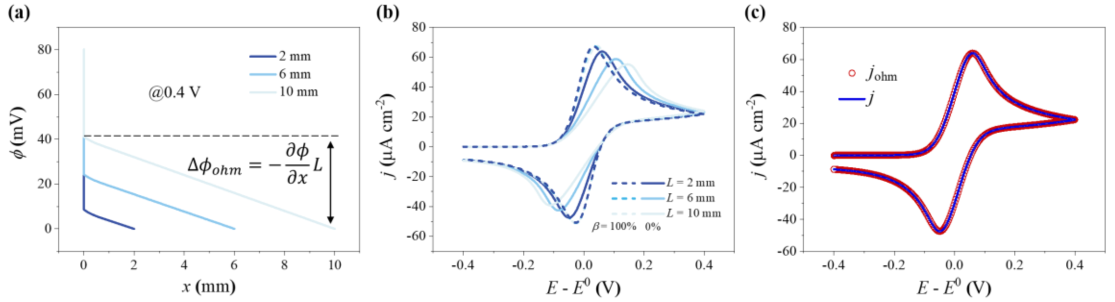

Figure S2. (a) The electrostatic potential distributions in the absence of the supporting electrolyte without compensation. (b) the influence of ohmic polarization in different length of solution (solid lines). The dashed lines are the corresponding CV responses with an ohmic compensation ratio of 100%. (c) the current (j_{ohm}) induced by ohm's law is equivalent to that of the total current (j).

As shown in Figure S2, the potential distribution consisting of two parts, including the interfacial EDL and the ohmic drop. The linear potential drop in the bulk solution increases with increasing solution length (L) in the absence of the supporting electrolyte, resulting in lower peak currents and lager peak separation. These results are widely recognized in the literature. [1-4] The ohmic polarization complicates the analysis of CV kinetics. It is necessary to perform ohmic compensation to eliminate the influence of solution resistance. The compensation is achieved by applying an additional potential corresponding to ohmic drop on the applied electrode potential. The overpotential is corrected by

$$\eta = E - E^0 - \phi_{\mathrm{H}} + \beta\Delta\phi_{ohm}, \quad \text{(S8)}$$

the first three terms on the right-hand side of the equation constitute the Frumkin corrected overpotential. $\Delta\phi_{ohm}$ is additional potential corresponding to ohmic drop. β is the compensation ratio. In our model, $\Delta\phi_{ohm}$ is self-consistently calculated by

$$\Delta\phi_{ohm} = -\left(\frac{\partial\phi}{\partial x}\right)_{x=L} L, \quad \text{(S9)}$$

where the potential gradient is given by that at the right boundary. The potential distribution in the bulk solution is linear. Stevens et al. have considered the perturbation of the concentration in the diffusion layer on the potential distribution. [4] The linear assumption remains valid in the region far from the electrode. We use Ohm's law to calculate the current

$$j_{ohm} = \frac{\Delta\phi_{ohm}}{R_s}, \tag{S10}$$

$$R_s = \frac{L}{\sum_i \frac{z_i^2 c_i F^2 D_i}{RT}}, \tag{S11}$$

where R_s is the solution resistance. As shown in Figure S2c, j_{ohm} is consistent with the total current, which verifies that the assumption of linear potential distribution is correct.

As illustrated in Figure S2b, the compensation level β uses 100%, resulting in increased peak currents and a narrower peak separation. From the results in Figure 3a in the main text, when β = 100%, the peak positions of CV response are consistent with those of the diffusion model. The ohmic polarization is thereby eliminated.

Mehandzhiyski et al. proposed that the reference boundary of electrostatic potential is set according to the actual spatial separation between the working electrode and the reference electrodes. However, as shown in Figure S2b, in the case of completely compensation (β = 100%), the CV responses are independent on the value of L. The influence of the length of the solution is no longer significant.

S3: The influence of the kinetic constant on the CV curves in the pure diffusion model

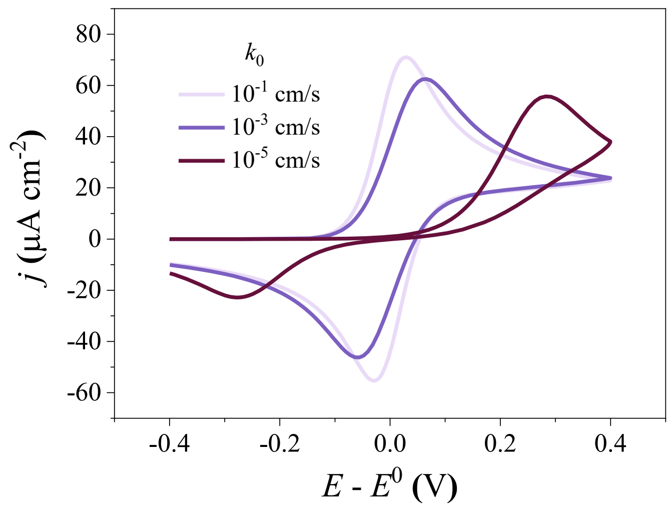

Figure S3. The influence of the kinetic constant on profiles of the CV curves in the pure diffusion model.

To avoid complications arising from the electrostatic interactions and isolate the impact of the kinetic constant on the CV response, the CV model is simplified to a diffusion model.

Most studies report that the kinetic constant is influenced by the identity and concentration of the cations of the supporting electrolyte. The reported kinetic constants fall in the range of 0.001 ~ 0.1 cm/s, [5-8] and the corresponding CV curves exhibit quasi-reversible behavior. As shown in Figure S3, the decreased kinetics produce decreased peak current and enhanced peak separation, while the CV curves remain the equal peaks.

However, in the experimental results, the anodic peak shifts positively, while the cathodic peak is almost completely absent. This indicates that the reduction process is significantly more polarized than the oxidation. Notably, even when the simulated kinetic constant is further decreased to 10^{-5} cm/s, resulting in a high polarized peak, the cathodic peak is still present.

The above discussion demonstrates that the kinetic constant is not the primary factor responsible for the unequal peaks in CV curves.

S4: Spatiotemporal distributions of the electrostatic potential and the concentration

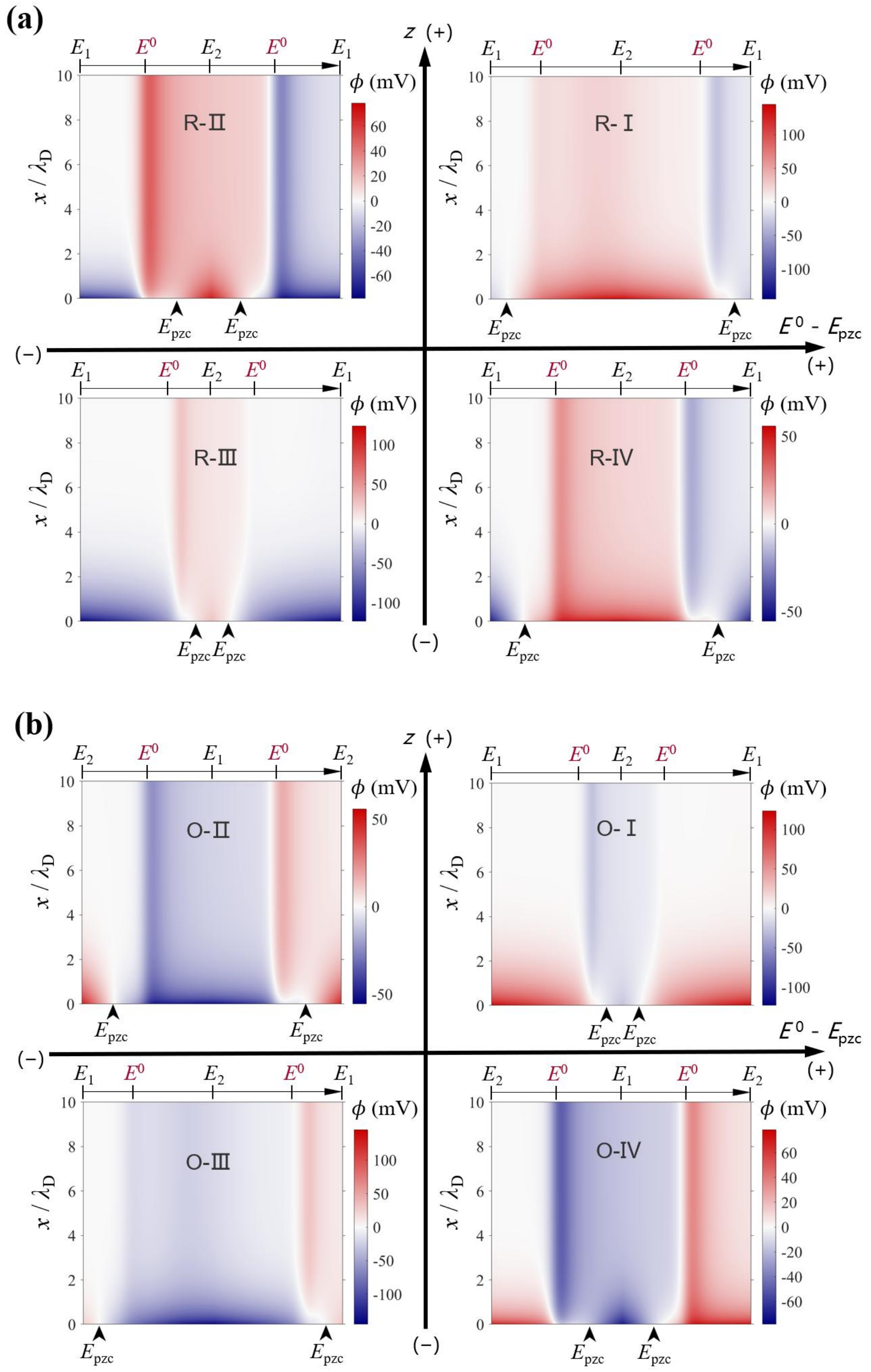

Figure S4. Spatiotemporal distributions of the electrostatic potential (ϕ) in the electrolyte without supporting salt. Corresponding cases are shown in Figure 5 of the main text. The initial reactant is (a) reductant, (b) oxidant.

As discussed in Figure 5 of the main text, the position of the PZC, the charge of the active ions, and the identity of the initial reactant species all influence the CV curves. Figure S4 illustrates the differences in the electrostatic potential distribution during the potential scanning under these conditions.

Focusing on the EDL region ($0 < x < 6\lambda_D$), the electrostatic potential is strongly governed by the surface charge of the electrode. When $E < E_{pzc}$, the electrostatic potential within the EDL is negative, whereas when $E > E_{pzc}$, it becomes positive. Therefore, the position of the formal potential (E^0) relative to the E_{pzc} determines the surface charge of the electrode at the moment of reaction. Further considering the charge of the ions (z), the electrode–ions interaction can be inferred as follows: Cases R-II, R-IV, O-II, and O-IV correspond to the attractive interaction, while cases R-I, R-III, O-I, and O-III correspond to the repelling interaction.

In the transition region ($6\lambda_D < x < 10\lambda_D$) between the EDL and the diffusion layer, the potential gradient is controlled by Ohm's law. When the anodic peak is generated, the potential gradient in the solution beyond $6\lambda_D$ becomes negative, corresponding to the positive potential in the transition region. Conversely, when a cathodic current occurs, the potential gradient is positive, corresponding to the negative potential in the transition region.

The difference between Figure S4a and S5b lies in the identity of the initial species, which determines the initial direction of the potential scanning. In Figure S4, the initial species is the reductant. The potential initially sweeps anodically for oxidation and then cathodically for reduction. Consequently, the electrostatic potential in the transition region in Figure S4a initially becomes positive and then transitions to negative. In contrast, the initial species in Figure S4b is the oxidant, the potential starts the scanning in a cathodic direction for reduction, and reverses for oxidation. As a result, the potential in the transition region first becomes negative and subsequently transitions to positive.

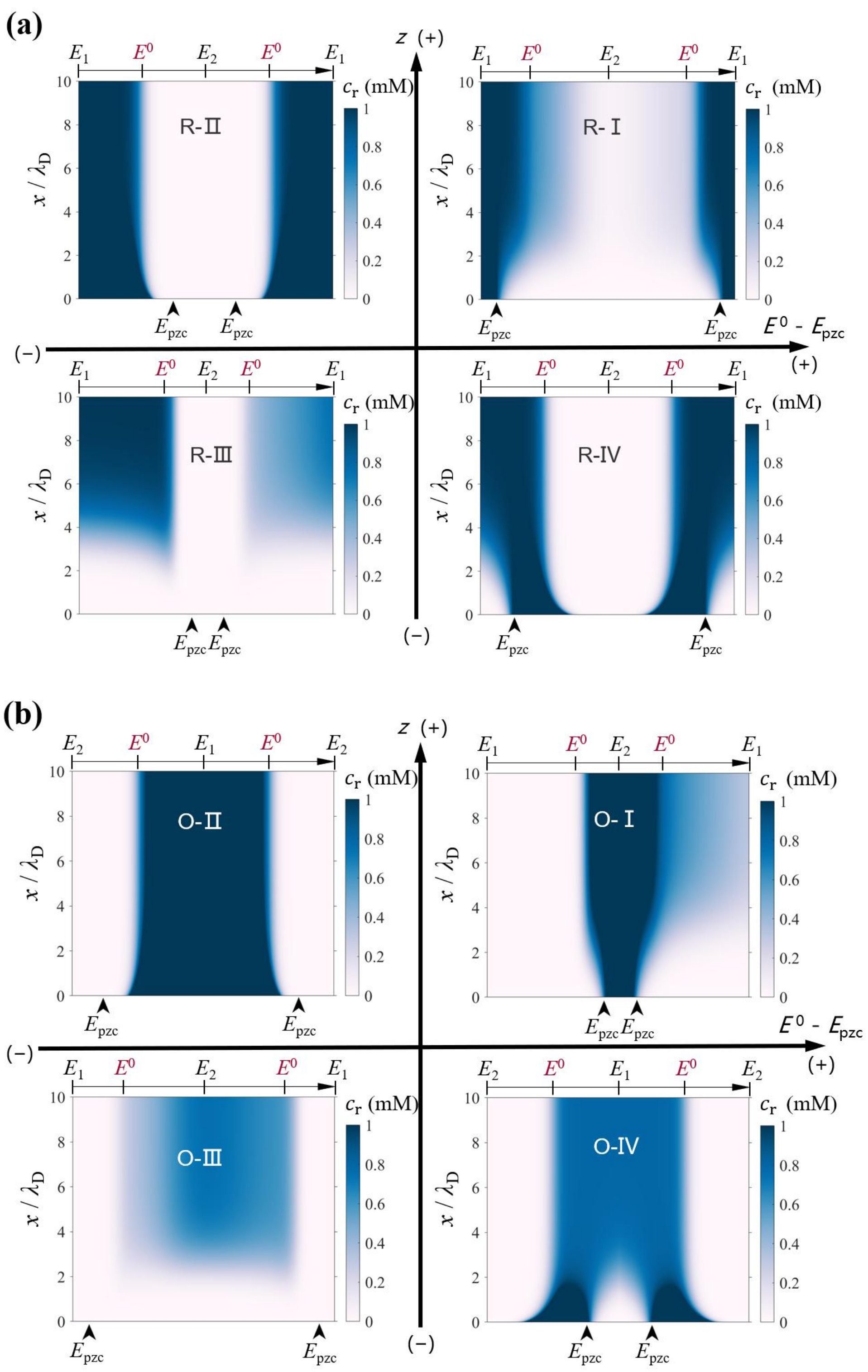

Figure S5. Spatiotemporal distributions of the reductant concentration (c_r). Corresponding cases are shown in Figure 3 of the main text. Corresponding cases are shown in Figure 3 of the main text. The initial reactant is (a) reductant, (b) oxidant.

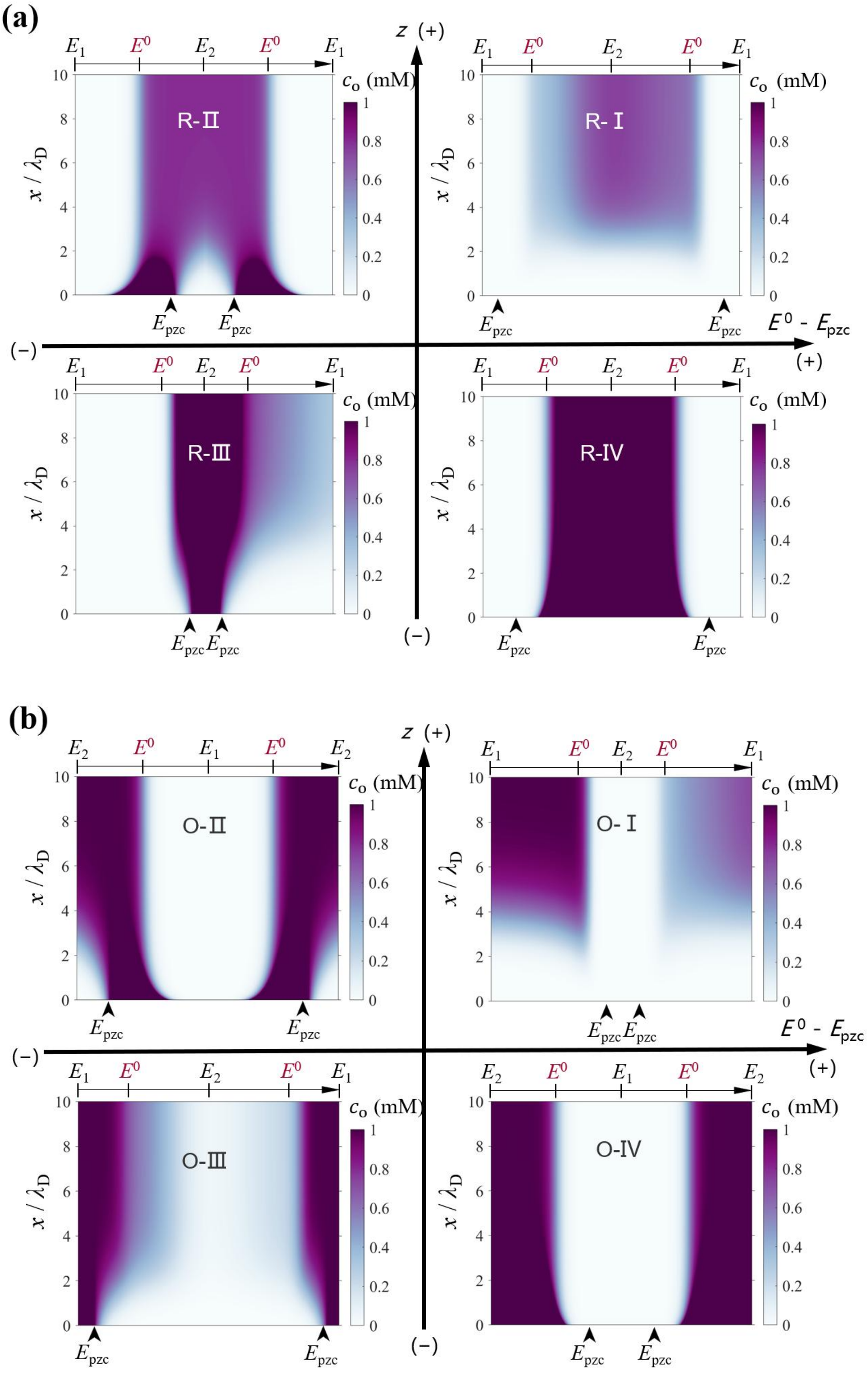

Figure S6. Spatiotemporal distributions of the oxidant concentration (c_{O}). Corresponding cases are shown in Figure 3 of the main text. Corresponding cases are shown in Figure 3 of the main text. The initial reactant is (a) reductant, (b) oxidant.

According to the analysis of the electrostatic potential in Figure S4, cases R-II, R-IV, O-II, and O-IV correspond to the attractive interaction, while cases R-I, R-III, O-I, and O-III correspond to the repelling interaction. In Figures S6 and S7, when the applied potential E is around E^0, the concentrations of the reductant (c_r) and the oxidant (c_o) at the electrode surface decrease in all attractive interaction scenarios, whereas they exceed the bulk values in all repulsive cases. The concentrations inside EDL regulate the reaction kinetics.

In all cases involving attractive interaction, the concentration profiles are symmetric with respect to the potential axis, while in the cases of the repulsive interaction, the profiles are asymmetric. The size of unequal peaks arises because repulsive interactions slow down the consumption of active species. In other words, the reactants cannot be fully depleted, resulting in the residual concentration features in the transition region. This behavior is reflected in the CV curves, where the oxidation and reduction peaks become unequal.

S5: The ratios of cathodic to anodic peaks and peak separations

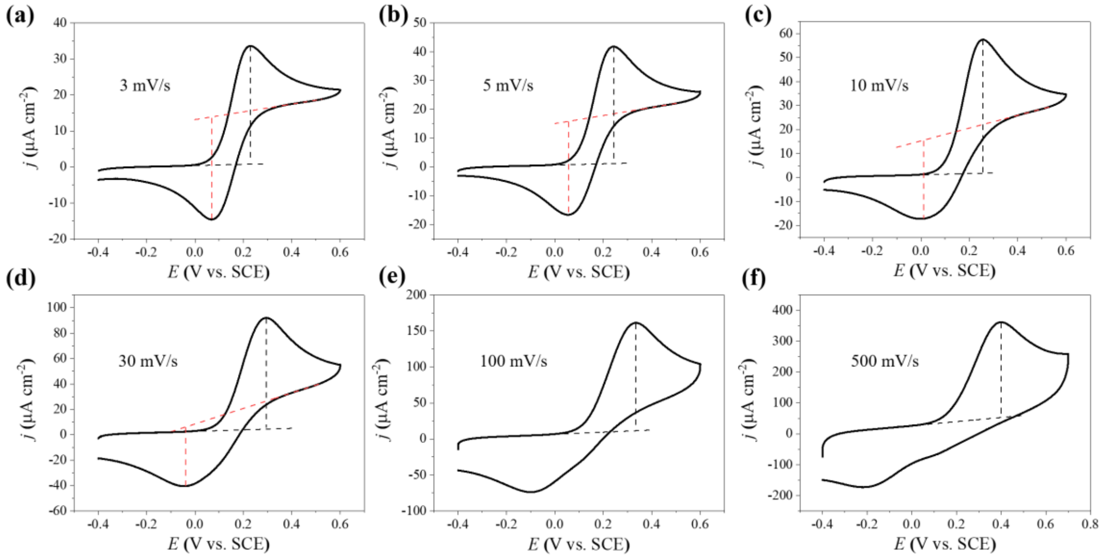

Figure S7. Experimental CV curves in 1 mM $K_4Fe(CN)_6$ + 10 mM $NaClO_4$ aqueous solution at different scanning rates (solid lines), the dashed lines are the baselines of the peaks.

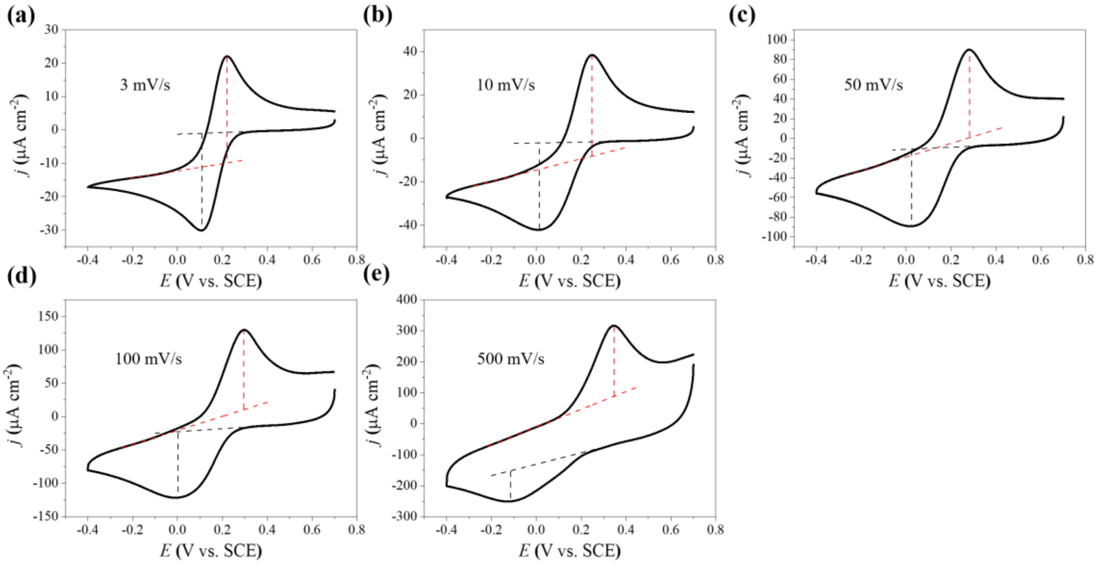

Figure S8. Experimental CV curves in 1 mM $K_3Fe(CN)_6$ + 10 mM $NaClO_4$ aqueous solution at different scanning rates (solid lines), the dashed lines are the baselines of the peaks.

Table S1. The peak currents, ratios of the cathodic to anodic peak currents, and peak separations at different scanning rate in 1 mM $K_4[Fe(CN)_6]$ + 10 mM $NaClO_4$ aqueous solution.

scanning rate (mV/s)	$j_{p,a}$ $(\mu A/cm^2)$	$j_{p,c}$ $(\mu A/cm^2)$	$j_{p,c}/j_{p,a}$	ΔE_p (mV)
3	33.6	28.5	0.85	156
5	41.8	32.5	0.78	186

10	55.8	32.8	0.59	244
30	87.8	46.4	0.53	338
100	149.9	49.4	0.33	429
500	309.5	/	/	612

Table S2. The peak currents, ratios of the cathodic to anodic peak currents, and peak separations at different scanning rate in 1 mM $K_3[Fe(CN)_6]$ + 10 mM $NaClO_4$ aqueous solution.

scanning rate (mV/s)	$j_{\mathrm{p,a}}$ $(\mu A/cm^2)$	$j_{\mathrm{p,c}}$ $(\mu A/cm^2)$	$j_{\mathrm{p,c}}/j_{\mathrm{p,a}}$	ΔE_{p} (mV)
3	49.0	44.8	0.91	113
10	46.2	39.6	0.86	237
50	39.4	35.0	0.88	259
100	36.4	29.6	0.81	292
500	32.4	13.9	0.42	462

As shown in Figure S7, the values of the anodic and cathodic peaks were obtained after background current correction. All peak currents and peak separations are summarized in Table S1. It should be noted that when the scanning rate exceeds 100 mV s^{-1}, the cathodic peaks are polarized toward large overpotentials. The subtraction of the background current may not be appropriate during the reverse sweep. This may lead to an underestimation of the reduction peak current. When the scanning rate falls in the range of 3~100 mV s^{-1}, the ratios of $j_{\mathrm{p,c}}/j_{\mathrm{p,a}}$ decrease with increasing scanning rate, indicating an enhanced dynamic EDL effect. Similarly, the peak current ratio in the $K_3[Fe(CN)_6]$ solution decreases as the scanning rate increases. As shown in the Figure S9 and Table S2.

S6: Step-by-step tutorial of implementation of the model in COMSOL

1. Building a new model as following operations,

Model Wizard >> **1D.**

2. Add two physical interfaces according to the following path:

Mathematics / PDE Interfaces / Coefficient Form PDE

3. click "**Done**" button, save the file as "CV_EDL".
4. **CV_EDL (root)** >> Set option "**Unit System**" to "**None**". This model performs dimensionless calculations.
5. Definition of parameters: Right click "**Global Definitions**" >> **Parameters** >> rename "**Label**" as "electrolyte parameters" >> click "**Load from File**" to import parametric file "electrolyte parameters.txt" >> Repeat the above operation to import file "electrode parameters.txt" and "constant.txt". Parametric files see Appendix 1.
6. Establishing the geometry: Right click "**Geometry 1**" >> **Interval** >> settings as follows >> click "**Build All Objects**" button. The whole space is divided into three segments, **1, 2 ,3**, respectively.

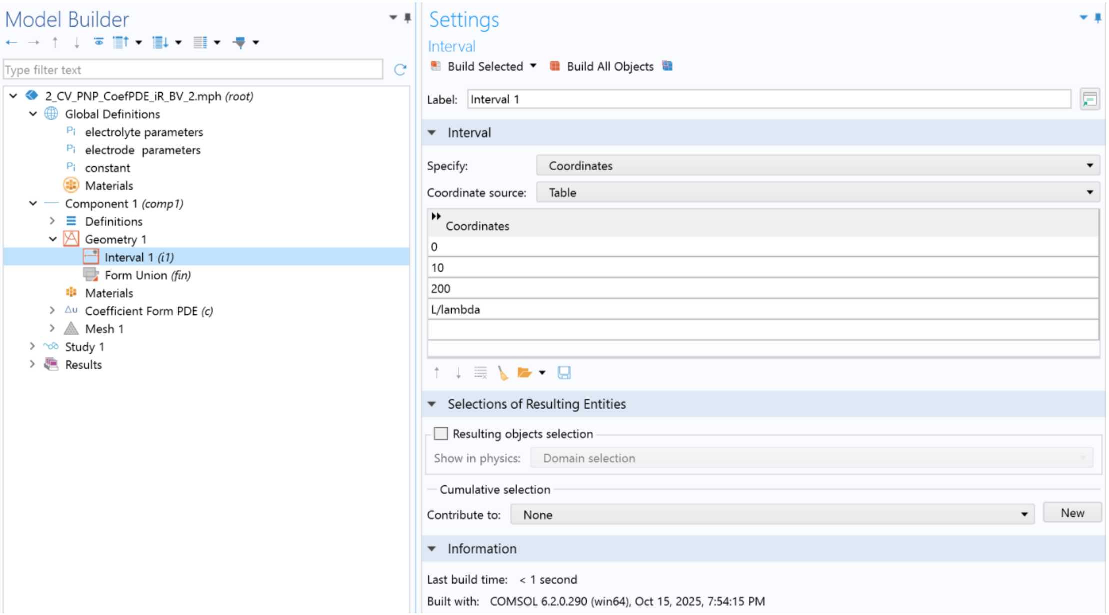

7. Discretization of mesh:
   7.1 Right click "**Mesh**" >> **Edge** >> set "**Geometric entity level**" to **Domain** >> Selection is line Region **1**;
   7.2 Right click "**Edge 1**" >> **Size** >> select "**Custom**" >> tick "**Maximum element size**" >> input "**0.1**";
   7.3 According to step 7.1 and 7.2, creating **Edge 2** and **Edge 3**. For **Edge 2** and **Edge 3**, select Region **2** and Region **3**, respectively, and set "**Maximum element size**" to **10** and **5e3**, respectively. Settings as follows. Click "**Build All**".
8. Defining variables, probes, and function calculation under **Component 1**

8.1 Right click “**Definitions**” >> **Variables** >> click “**Load from File**” to import variables file. “variables_EGV.txt” for EGV kinetics, “variables_MHC.txt” for MHC kinetics;

8.2 Right click “**Definitions**” >> **Waveform** >> rename “**Function name**” as **phi_M** >> settings as follow. This function is used to apply triangular waves of electrode potential;

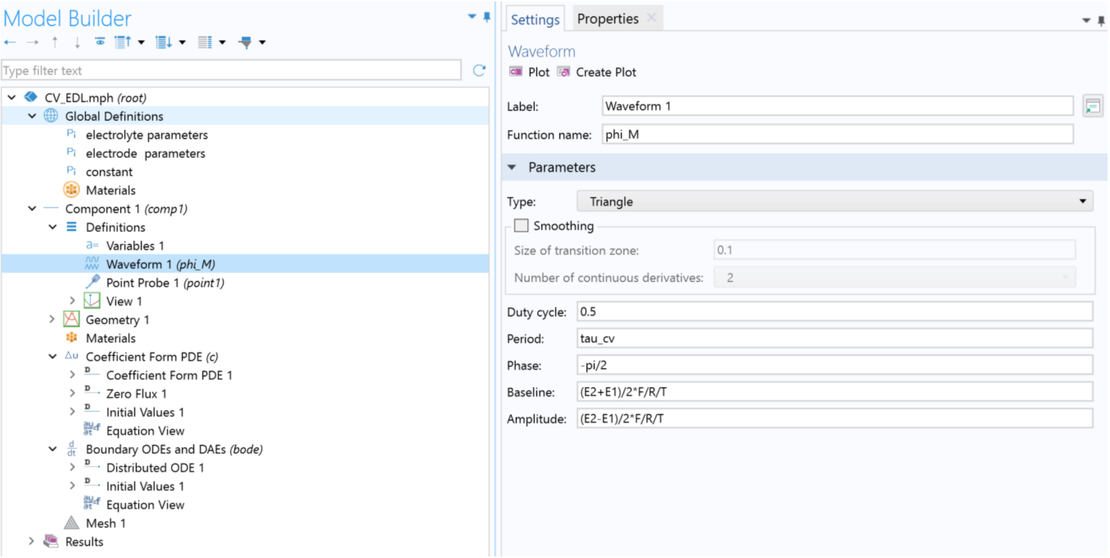

8.3 Right click “**Definitions**” >> **Probes** >> **Point Probe** >> rename “**Label**” as **potential gradient** >> set “**selection**” to Boundary **4** >> input **Phix** into “**Expression**”;

9. Settings of partial differential equation:

9.1 select “**Coefficient Form PDE (c)**” >> set “**Selection**” to **All domains** >> set “**Number of dependent variables**” to **5** >> **Dependent variables** are successively modified to “**C_o**”, “**C_r**”, “**C_p**”, “**C_n**”, and “**Phi**”;

9.2 select “**Coefficient Form PDE 1**” >> input the **Diffusion Coefficient** (**c**), **Absorption Coefficient** (**a**), and **Damping of Mass Coefficient** (**d_a**) as following settings. The other coefficients are zero.

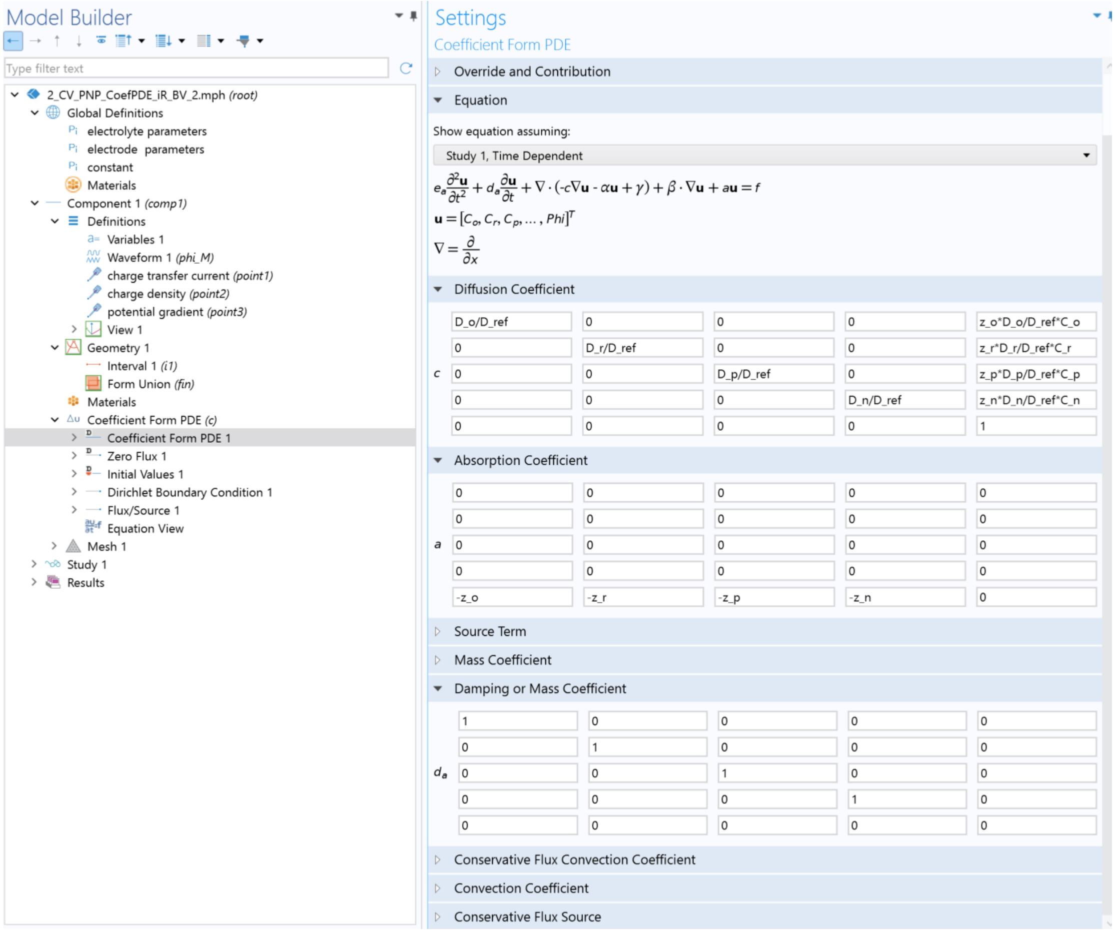

Here are some annotations. The PNP equation is

$$\frac{\partial c_i}{\partial t} = \frac{\partial}{\partial x}\left[D_i \frac{\partial c_i}{\partial x} + D_i \frac{z_i c_i F}{RT}\frac{\partial \phi}{\partial x}\right],$$

$$\frac{\partial}{\partial x}\left(\epsilon \frac{\partial \phi}{\partial x}\right) = -F\sum_i z_i c_i,$$

the dimensionless form of the PNP equation is

$$\frac{\partial C_i}{\partial \tau} = \frac{\partial}{\partial X}\left[\bar{D}_i \frac{\partial C_i}{\partial X} + \bar{D}_i z_i C_i \frac{\partial \Phi}{\partial X}\right],$$

$$\frac{\partial}{\partial X}\left(\frac{\partial \Phi}{\partial X}\right) = -\sum_i z_i C_i,$$

where $\Phi = \phi\frac{F}{RT}$ is dimensionless potential. $C_i = \frac{c_i}{c_{ref}}$ is the dimensionless concentration of i species, with $c_{ref} = z_o^2 c_o^b + z_r^2 c_r^b + z_p^2 c_p^b + z_n^2 c_n^b$ being the reference concentration. c_o^b, c_r^b, c_p^b, and c_n^b are the bulk concentrations of the oxidant, reductant, cations and anions in the

supporting electrolyte, respectively. z_o, z_r, z_p, and z_n are corresponding charge. $\tau = t\frac{D_{ref}}{\lambda^2}$ is the dimensionless time, with $\lambda = \sqrt{\frac{\epsilon RT}{F^2 c_{ref}}}$ being the Debye length, $D_{ref} = 5 \times 10^{-10}\ m^2/s$ reference diffusion coefficient. $\overline{D_i} = \frac{D_i}{D_{ref}}$ is the dimensionless diffusion coefficient of i species. X is normalized spatial coordinate, $\frac{x}{\lambda}$.

The PNP equation is rewritten as a matrix form,

$$d_a \frac{\partial u}{\partial \tau} + \nabla \cdot (-c\nabla u) + au = 0$$

where

$$u = \begin{bmatrix} C_o \\ C_r \\ C_p \\ C_n \\ \Phi \end{bmatrix}$$

$$d_a = \begin{bmatrix} 1 & 0 & 0 & 0 & 0 \\ 0 & 1 & 0 & 0 & 0 \\ 0 & 0 & 1 & 0 & 0 \\ 0 & 0 & 0 & 1 & 0 \\ 0 & 0 & 0 & 0 & 0 \end{bmatrix}$$

$$a = \begin{bmatrix} 0 & 0 & 0 & 0 & 0 \\ 0 & 0 & 0 & 0 & 0 \\ 0 & 0 & 0 & 0 & 0 \\ 0 & 0 & 0 & 0 & 0 \\ -z_o & -z_r & -z_p & -z_n & 0 \end{bmatrix}$$

$$c = \frac{1}{D_{\mathrm{ref}}} \begin{bmatrix} D_o & 0 & 0 & 0 & z_o D_o C_o \\ 0 & D_r & 0 & 0 & z_r D_r C_r \\ 0 & 0 & D_p & 0 & z_p D_p C_p \\ 0 & 0 & 0 & D_n & z_n D_n C_n \\ 0 & 0 & 0 & 0 & 1 \end{bmatrix}$$

9.3 select "**Initial Values 1**" >> input "**c_o_bulk/c_ref**", "**c_r_bulk/c_ref**", "**c_p_bulk/c_ref**", "**c_n_bulk/c_ref**" into initial value for C_o, C_r, C_p, C_n, respectively.

9.4 Right click "**Coefficient Form PDE (c)**" >> **Dirichlet Boundary Condition** >> set "**Selection**" to Boundary **4** >> input "**c_o_bulk/c_ref**", "**c_r_bulk/c_ref**", "**c_p_bulk/c_ref**", "**c_n_bulk/c_ref**" into prescribed value of C_o, C_r, C_p, C_n, respectively.

9.5 Right click "**Coefficient Form PDE (c)**" >> **Flux/Source** >> set "**Selection**" to

Boundary **1** >> set coefficients as following settings,

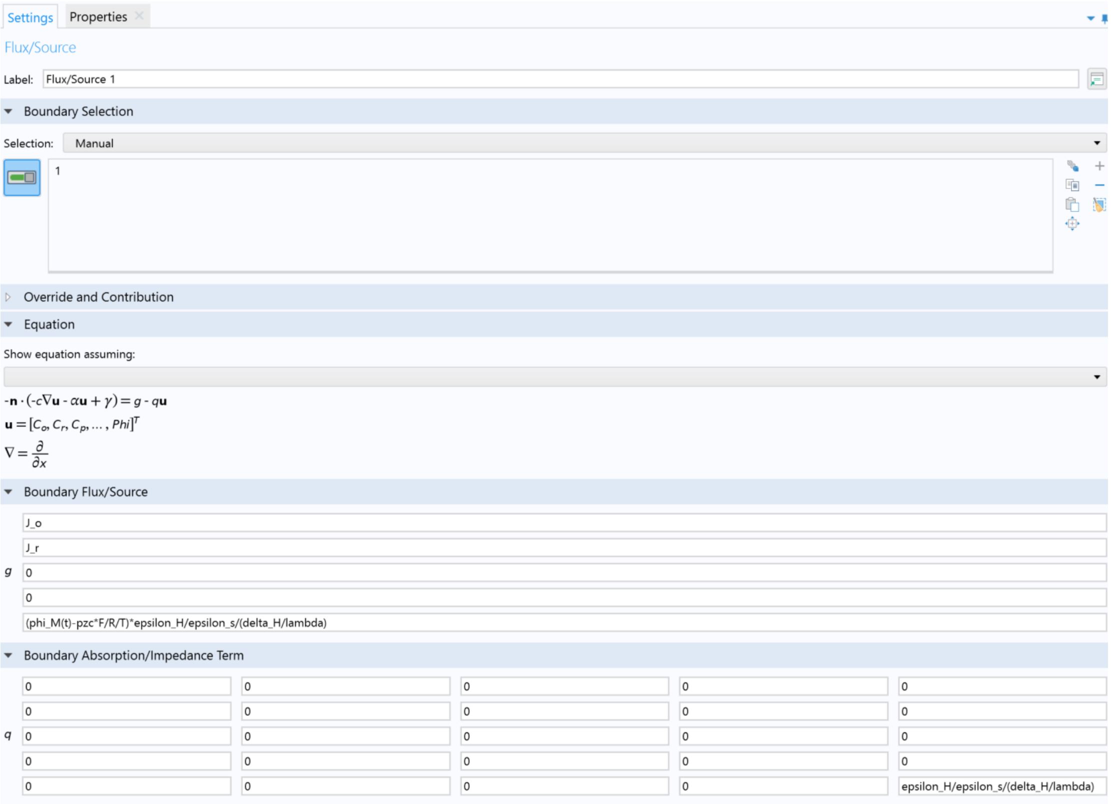

10. Add study and solver configuration:
    10.1 Click “**Add Study**” >> double-click “**Time Dependent**”;
    10.2 select “**Step 1: Time Dependent**” >> Output times: range(0, tau_cv/500, tau_cv) >> set “Tolerance” to **User controlled** >> Relative tolerance: 0.001;
    10.3 Right click “**Step 1: Time Dependent**” >> **Show default solver** >> select **Time-Dependent Solver 1** according to the path: Solver Configuration / Solution 1/ Time-Dependent Solver 1 >> In “**General**” window, set “**Times to store**” to “**Steps taken by solver**” >> In “**Time Stepping**” window, tick “**Initial step**”, and set to **1e-5**; set “**Maximum step constraint**” as **Constant**, and input **tau_cv/500** into “**Maximum step**”
    10.4 Select **Fully Coupled 1**, according to the path: Solver Configuration / Solution 1/ Time-Dependent Solver 1 / Fully Coupled 1 >> In “**Method and Termination**” window, select **Automatic (Newton)** as the option of the “**Nonlinear method**”.
11. Click “**Compute**” button to run model.
12. Plot: Right click “**Results**” >> **1D Plot Group** >> right click “**1D Plot Group 1**” >> **Point Graph** >> in “**y-Axis Data**” window, “**Expression**” is **j_total** >> in “**x-Axis Data**” window, “**Expression**” is **phi_M(t)*R*T/F**.

Appendix 1

Electrolyte parameters:

c_ref z_o^2*c_o_bulk+z_r^2*c_r_bulk+z_p^2*c_p_bulk+z_n^2*c_n_bulk "reference concentration"

z_r -4 "charge number of the reductant"

z_p 1 "charge number of the positive ions"

z_o z_r+n "charge number of the oxidant"

z_n -1 "charge number of the negative ions"

Rs L*R*T/F^2/(z_o^2*c_o_bulk*D_o+z_r^2*c_r_bulk*D_r+z_p^2*c_p_bulk*D_p+z_n^2*c_n_bulk*D_n) "[ohm m^2] solution resistance"

c_sp 0 "[mol/m^3] bulk concentration of the supporting electrolyte"

c_p_bulk "c_sp - (1-sign(z_r))/2*z_r*c_r_bulk - (1-sign(z_o))/2*z_o*c_o_bulk" "[mol/m^3] bulk concentration of the cation of the supportting electrolyte"

c_n_bulk "c_sp + (1+sign(z_r))/2*z_r*c_r_bulk + (1+sign(z_o))/2*z_o*c_o_bulk" "[mol/m^3] bulk concentration of the anion of the supportting electrolyte"

c_r_bulk 1 "[mol/m^3] bulk concentration of reductant"

c_o_bulk 0 "[mol/m^3] bulk concentration of oxidant"

delta_H 6e-10 "[m] thickness of the Helmholtz layer"

L 0.002 "[m] right boundary"

lambda sqrt(epsilon_s*R*T/c_ref/F^2) "[m] Debye length"

D_ref 7e-10 "[m^2/s] reference diffusion coefficient"

D_r 7e-10 "[m^2/s] diffusion coefficient of reductant"

D_o 7e-10 "[m^2/s] diffusion coefficient of oxidant"

D_p 2e-9 "[m^2/s] diffusion coefficient of cations of the supporting electrolyte"

D_n 2e-9 "[m^2/s] diffusion coefficient of anions of the supporting electrolyte"

epsilon_s 78.5*epsilon0_const "[F/m] dielectric constant of the solvent"

epsilon_H 4*epsilon0_const "[F/m] dielectric constant of the Helmholtz layer"

lamb 1.2 "[eV] reorgnization energy"

Electrode parameters:

alpha 0.5 "transfer coefficient"

E0 0 "[V] formal potential"

v 0.01 "[V/s] scanning rate"

E1 -0.6 "[V] lower limit potential"

E2 0.3 "[V] upper limit potential"

t_cv 2*abs(E1-E2)/v "[s] time of one cycle"

tau_cv t_cv*D_ref/lambda^2 "dimensionless time of one cycle"

pzc 0.2 "[V] potential of zero charge"

Constant

k0 1e-3 "[m/s] standard rate constant"

n 1 "electron transfer number"

T 298.15 "[T] temperature"

R 8.314 "[J/mol/K] gas constant"

F 96485 "[C/mol] Faraday constant"

beta 1 "compensation level"

k_B R/N_A_const "[J/K] Boltzmann constant"

e0 F/N_A_const "[C] elementary charge"

bd 1 "[eV] integral boundary of energy"

variables_EGV

eta "phi_M(t)-Phi-E0*F/R/T- beta*point1*(L/lambda)" "dimensionless effective overpotential"

j_ct "n*F*k0*c_ref*(C_r*exp((1-alpha)*eta) - C_o*exp(-alpha*eta))" "charge transfer current"

J_o "(k0*lambda/D_ref)*(C_r*exp(n*(1-alpha)*eta) - C_o*exp(n*(-alpha)*eta))" "dimensionless flux of oxidant"

J_r -J_o "dimensionless flux of reductant"

j_total "j_ct + d(sigma_M,t)*D_ref/lambda^2" "total current"

sigma_M -epsilon_s*Phix*(R*T/F/lambda) "surface charge density"

variables_MHC

eta "phi_M(t)-Phi-E0*F/R/T- beta*point1*(L/lambda)" "dimensionless effective overpotential"

j_ct "n*F*D_ref*c_ref/lambda*(K_ox*C_r - K_red*C_o)" "charge transfer current"

J_o "K_ox*C_r - K_red*C_o" "dimensionless oxidant flux"

J_r -J_o "dimensionless reductant flux"

j_total "j_ct + d(sigma_M,t)*D_ref/lambda^2" "total current"

K_ox (k0*lambda/D_ref)*Omega_ox/Omega_ox_0 "dimensionless oxidation rate"

K_red (k0*lambda/D_ref)*Omega_red/Omega_red_0 "dimensionless reduction rate"

Omega_ox "integrate((1-1/(1+exp(f*e0/k_B/T)))*exp(-(f*e0-eta*k_B*T+lamb*e0)^2/4/k_B/T/(lamb*e0)), f, -bd, bd)" "oxidation rate"

Omega_ox_0 "integrate((1-1/(1+exp(f*e0/k_B/T)))*exp(-(f*e0+lamb*e0)^2/4/k_B/T/(lamb*e0)), f, -bd, bd)" "oxidation rate when eta = 0"

Omega_red "integrate(1/(1+exp(f*e0/k_B/T))*exp(-(f*e0-eta*k_B*T-lamb*e0)^2/4/k_B/T/(lamb*e0)), f, -bd, bd)" "reduction rate"

Omega_red_0 "integrate(1/(1+exp(f*e0/k_B/T))*exp(-(f*e0-lamb*e0)^2/4/k_B/T/(lamb*e0)), f, -bd, bd)" "reduction rate when eta = 0"

sigma_M -epsilon_s*Phix*(R*T/F/lambda) "surface charge density"

Reference

1. Dickinson, E. J. F.; Limon-Petersen, J. G.; Rees, N. V.; Compton, R. G., How Much Supporting Electrolyte Is Required to Make a Cyclic Voltammetry Experiment Quantitatively "Diffusional"? A Theoretical and Experimental Investigation. *The Journal of Physical Chemistry C* **2009,** *113* (25), 11157-11171.

2. Belding, S. R.; Compton, R. G., Cyclic voltammetry in the absence of excess supporting electrolyte: The effect of analyte charge. *J. Electroanal. Chem.* **2012,** *683*, 1-13.

3. Levey, K. J.; Edwards, M. A.; White, H. S.; Macpherson, J. V., Simulation of the cyclic voltammetric response of an outer-sphere redox species with inclusion of electrical double layer structure and ohmic potential drop. *Phys. Chem. Chem. Phys.* **2023,** *25* (11), 7832-7846.

4. Stevens, N. P. C.; Rooney, M. B.; Bond, A. M.; Feldberg, S. W., A Comparison of Simulated and Experimental Voltammograms Obtained for the [Fe(CN)6]3-/4- Couple in the Absence of Added Supporting Electrolyte at a Rotating Disk Electrode. *The Journal of Physical Chemistry A* **2001,** *105* (40), 9085-9093.

5. Kawiak, J.; Jędral, T.; Galus, Z., A reconsideration of the kinetic data for the $Fe(CN)_6^{3-}/Fe(CN)_6^{4-}$ system. *J. Electroanal. Chem. Interfacial Electrochem.* **1983,** *145* (1), 163-171.

6. Peter, L. M.; Dürr, W.; Bindra, P.; Gerischer, H., The influence of alkali metal cations on the rate of the Fe(CN)64−/Fe(CN)63− electrode process. *J. Electroanal. Chem. Interfacial Electrochem.* **1976,** *71* (1), 31-50.

7. Bindra, P.; Gerischer, H.; Peter, L. M., The dependence of the rate of the Fe(CN)63−/Fe(CN)64− couple on ionic strength in concentrated solutions. *J. Electroanal. Chem. Interfacial Electrochem.* **1974,** *57* (3), 435-438.

8. Kůta, J.; Yeager, E., The influence of cations on the electrode kinetics of ferricyanide-ferrocyanide system on the rotating gold electrode. *J. Electroanal. Chem. Interfacial Electrochem.* **1975,** *59* (1), 110-112.
